# Supplementary material for: Prevalence of sexual violence and its associated factors among housemaids attending evening schools in urban settings of Gedeo zone, Southern Ethiopia: A school based cross sectional study
Source: PLoS One. 2021 Oct 29;16(10):e0258953. doi: 10.1371/journal.pone.0258953 (PMC8555818; doi:10.1371/journal.pone.0258953)
Supplement: Attachment — Submitted filename: PONE-D-20-12624_R1 reviewer.pdf [file pone.0258953.s002.pdf]

PLOS ONE

Prevalence of sexual violence and its associated factors among housemaids attending evening schools in urban settings of Gedeo zone, Southern Ethiopia: A school based cross sectional study

--Manuscript Draft--

**Manuscript Number:**	PONE-D-20-12624R1
**Article Type:**	Research Article
**Full Title:**	Prevalence of sexual violence and its associated factors among housemaids attending evening schools in urban settings of Gedeo zone, Southern Ethiopia: A school based cross sectional study
**Short Title:**	Sexual violence among housemaids in Southern Ethiopia
**Corresponding Author:**	Mohammed Feyisso Shaka, MPH<br>Dilla University<br>Dilla, SNNP ETHIOPIA
**Keywords:**	sexual violence; housemaids; evening school; Gedeo zone
**Abstract:**	Background: Housemaids are the most vulnerable group to sexual violence due to their working condition, isolation and school arrangements. Despite the ubiquity of sexual violence among the domestic work sector, particularly among housemaids, this area of research continues to be neglected. This study was aimed at examining the prevalence and factors pertaining to sexual violence among housemaids attending night school program in urban setups of Gedeo zone, Southern Ethiopia.<br>Method : A school based quantitative cross sectional study was conducted among 394 housemaids attending night schools in the urban setups of Gedeo Zone from April to May 2019. After stratifying of students using class grade, SRS technique was used to choose study subjects from each stratum. Quantitative data was collected using face to face interview and qualitative was collected using focus group discussion, in-depth interview and key informant interview. The data was entered and analyzed by SPSS version 20. Binary logistic regression was fitted to determine the association of each independent variable with the dependent variable.<br>Result: Based on the finding, the prevalence of sexual violence was 60.2%. The odds of experiencing sexual violence through working life-time as housemaid was higher for those who had migrated from rural to urban for work [AOR =1.97: 95 % CI ,(1.07,3.63)], had less than 5 years of experience as housemaid [AOR = 3.10 : 95 % CI , (1.60 , 6.00)], were in the age group of 15-19 [AOR = 3.75 :95 % CI (1.88 ,7.46), ever used alcohol [AOR = 6.77 : 95 % CI,(2.65,17.33)] and whose fathers lacked formal education [AOR= 2.75 :95%CI (1.24,6.08)]. On the other hand, unmarried /housemaids having no regular sexual partner were less likely to face sexual violence [AOR. =0.28: 95% CI, (0.13, 0.57)].<br>Conclusion: The level of s exual violence was found to be high among housemaids attending night schools in in this study. Housemaids from rural area, those newly starting the work, younger housemaids, and those who were married were more likely to be victims of sexual violence.
**Order of Authors:**	Mohammed Feyisso Shaka, MPH
	Kalkidan Gezahegn
	Selamawit Semagn
**Response to Reviewers:**	Rebuttal Letter_Response to reviewers<br>PONE-D-20-12624<br>Prevalence of sexual violence and its associated factors among housemaids attending evening schools in urban setups of Gedeo zone, Southern Ethiopia: A school based cross sectional study<br>PLOS ONE<br>2. Please address the following:<br>- Please include additional information regarding the interview guide used in the study and ensure that you have provided sufficient details that others could replicate the analyses. For instance, if you developed a guide as part of this study and it is not under

Powered by Editorial Manager® and ProduXion Manager® from Aries Systems Corporation

a copyright more restrictive than CC-BY, please include a copy, in both the original language and English, as Supporting Information. In addition, please include the details of the pre-testing of this tool, i.e. the number of participants and where they were recruited from.
- Please refrain from stating p values as 0.00, either report the exact value or employ the format p<0.001.
Response: The detail about pretest was explained in the manuscript page four paragraph one. Additional information about the tool was included and due to copyright issue we couldn't include as supporting information. P-value was corrected accordingly.
3. You indicated that you had ethical approval for your study. In your Methods section, please ensure you have also stated whether you obtained consent from parents or guardians of the minors included in the study or whether the research ethics committee or IRB specifically waived the need for their consent.
Response: It is explained in the manuscript under Ethical consideration section, page 6, Paragraph 1.
4. We note that you have indicated that data from this study are available upon request. PLOS only allows data to be available upon request if there are legal or ethical restrictions on sharing data publicly. For information on unacceptable data access restrictions, please see http://journals.plos.org/plosone/s/data-availability#loc-unacceptable-data-access-restrictions.
In your revised cover letter, please address the following prompts:
a) If there are ethical or legal restrictions on sharing a de-identified data set, please explain them in detail (e.g., data contain potentially identifying or sensitive patient information) and who has imposed them (e.g., an ethics committee). Please also provide contact information for a data access committee, ethics committee, or other institutional body to which data requests may be sent.
b) If there are no restrictions, please upload the minimal anonymized data set necessary to replicate your study findings as either Supporting Information files or to a stable, public repository and provide us with the relevant URLs, DOIs, or accession numbers. Please see http://www.bmj.com/content/340/bmj.c181.long for guidelines on how to de-identify and prepare clinical data for publication. For a list of acceptable repositories, please see http://journals.plos.org/plosone/s/data-availability#loc-recommended-repositories.
We will update your Data Availability statement on your behalf to reflect the information you provide.
Response: We have uploaded the data on Dryad with DOI of https://doi.org/10.5061/dryad.hhmgqnkfv
5. Your ethics statement should only appear in the Methods section of your manuscript. If your ethics statement is written in any section besides the Methods, please delete it from any other section.
Response: Corrected accordingly. Page 6 paragraph 1
6. Please ensure that you refer to Figure 1 in your text as, if accepted, production will need this reference to link the reader to the figure.
Response: Corrected. Page 8
7. We note you have included a table to which you do not refer in the text of your manuscript. Please ensure that you refer to Table 1 and Table 2 in your text; if accepted, production will need this reference to link the reader to the Table.
Response: We have corrected the typing error regarding the table numbers
8. Please include a copy of Table 3 which you refer to in your text on page 6.

Reviewers' comments:

Reviewer's Responses to Questions
Comments to the Author

Reviewer #1: Many expressions of the author are not standard, including the display of the table. For example, when the author expresses the result, sometimes the numbers are expressed in Arabic numerals, sometimes in words, and some sentences are not clear. Please refer to the published article format and expression for revision.
Response: We have tried to make the presentation of the table to be to the standard. Unnecessary, numerical presentations in the table were modified
Reviewer #2: Does the manuscript adhere to the PLOS Data Policy? Additional details

can be found at http://journals.plos.org/plosone/s/materials-and-software-sharing. (Answer options: Yes, No

"I don't know"

1. It is obvious that the writing need to be improved. I suggest the authors ask for a help from someone whose native language is English.
Response: The manuscript English writing was proofread by Proffessional proofreaders to improve readability.
2. Section of "sample technique":
1) The author described that a random cluster sampling was used to choose the evening school and then assigned the study participants according to the class grades. We didn't find the class distribution of participants. Among the participants, "279 (70.8%) were adolescents (15-19 years old) whereas 115 (29.2%) were young adults (20- 24 years)". Does the unbalanced sample exist
Response: Class distribution of the students was briefly explained in the methodology under sampling technique subsection page 4. Regarding issue about existence of unbalanced sampling, the proportion is not due to unbalanced sampling. It entirely due to the actual age distribution of the population.
2) "random sampling technique was used", "data collectors were revisited the class room two times at different time intervals and for those who were absent after two revisits, the participant was replaced by another in the sampling frame". How many women were replaced? Does the replace strategy have any effects on the study results?
Response: The number of women who replaced for their absence was not significant where only three women were missed and replaced. Replacement was also made by randomly selecting the participant from the previously unselected eligible population.
3. Section of "measurement"
1) The sexual harassment is the most common of sexual violence experience for housemaid have experienced in this study. The author didn't explain sexual harassment in detail, has one appropriate joke related to sex been included? If this, the proportion of sexual violence might be overestimated.
Response: Explained under measurement subsection second paragraph page 5
2) The alcoholic use has not been evaluated semi-quantitatively or quantitatively. This is a common-sense question: What time and how much of the alcohols consumption is the most important for the victim suffer from sexual violence.
Response: The ASSIST tool is standard tool for assessment of the common substance uses based on the frequency and duration of use. We used that tool. With that tool we couldn't find any women fulfilling criteria for substance use. So we measure the episodes of occasional use and classified alcohol use as ever use or not at all
4. Section of "results":
1) In the descriptive analysis section, the author only needs to reveal the proportion of some important variables, for example those covariates lead to significant changes in the logistic model or other characteristic variables related to sexual violent founded from previous study.
Response: We have tried to remove some variables thought to be less important to present in this study. However, as one of the objective of this study is to describe the violence and the characteristics of the study participants, we retained most of the variables thought to have relevant descriptive information.
2) Are the forms of the tables' correct? Please refer to the author guidance of PLOS ONE.
5. Section of "Discussion"
1) The author discussed the results in comparability instead of analysis.
Response: We have tried to present with the analysis of the findings
2) "This might be due to…", "The possible explanation for this finding…", "The possible suggestion might be…", "This result might be due to …" were used by authors to explain the association between the related factors and sexual violence. It is suspicious that the author's knowledge for the study field or the amounts of reference the author has read.
Response: We have modified our way of explanation accordingly
3) It is interesting for the findings that the educational attainments of fathers were associated with the sexual violence for the housemaids suffered. A health education system should be considered to help establishing an intimate father-daughter relationship in these districts.

	Response: The finding of the study was disseminated to the local health planners and this will be an input to address the issue.<br><br>____<br>Thank you for your interesting and valuable comments that made us to improve our work substantially.
**Additional Information:**	
**Question**	**Response**
**Financial Disclosure**<br><br>Enter a financial disclosure statement that describes the sources of funding for the work included in this submission. Review the submission guidelines for detailed requirements. View published research articles from *PLOS ONE* for specific examples.<br><br>This statement is required for submission and **will appear in the published article** if the submission is accepted. Please make sure it is accurate.<br><br>**Unfunded studies**<br>Enter: *The author(s) received no specific funding for this work.*<br><br>**Funded studies**<br>Enter a statement with the following details:<br>• Initials of the authors who received each award<br>• Grant numbers awarded to each author<br>• The full name of each funder<br>• URL of each funder website<br>• Did the sponsors or funders play any role in the study design, data collection and analysis, decision to publish, or preparation of the manuscript?<br>• **NO** - Include this sentence at the end of your statement: *The funders had no role in study design, data collection and analysis, decision to publish, or preparation of the manuscript.*<br>• **YES** - Specify the role(s) played.<br><br>* typeset	Funding for this study was covered by Dilla University NORHED/SENUPH project and the funding organization have no any role in methodology, analysis, interpretation, and write up of the manuscript
**Competing Interests**<br><br>Use the instructions below to enter a competing interest statement for this submission. On behalf of all authors, disclose any competing interests that	The authors have declared that no competing interests exist.

could be perceived to bias this work—acknowledging all financial support and any other relevant financial or non-financial competing interests.<br><br>This statement **will appear in the published article** if the submission is accepted. Please make sure it is accurate. View published research articles from *PLOS ONE* for specific examples.<br><br>**NO authors have competing interests**<br><br>Enter: *The authors have declared that no competing interests exist.*<br><br>**Authors with competing interests**<br><br>Enter competing interest details beginning with this statement:<br><br>*I have read the journal's policy and the authors of this manuscript have the following competing interests: [insert competing interests here]*<br><br>* typeset	
**Ethics Statement**<br><br>Enter an ethics statement for this submission. This statement is required if the study involved:<br><br>• Human participants<br>• Human specimens or tissue<br>• Vertebrate animals or cephalopods<br>• Vertebrate embryos or tissues<br>• Field research<br><br>Write "N/A" if the submission does not require an ethics statement.<br><br>General guidance is provided below. Consult the submission guidelines for detailed instructions. **Make sure that all information entered here is included in the Methods section of the manuscript.**	Ethical clearance was obtained from Institutional Review Board of Dilla University, College of Health Science and Medicine. Written informed consent was obtained from each respondent after detailed explanation of the nature and procedure of the study. The information given by each respondent was kept with strict confidentiality and name was not recorded.

Powered by Editorial Manager® and ProduXion Manager® from Aries Systems Corporation

**Format for specific study types**<br><br>**Human Subject Research (involving human participants and/or tissue)**<br>• Give the name of the institutional review board or ethics committee that approved the study<br>• Include the approval number and/or a statement indicating approval of this research<br>• Indicate the form of consent obtained (written/oral) or the reason that consent was not obtained (e.g. the data were analyzed anonymously)<br><br>**Animal Research (involving vertebrate animals, embryos or tissues)**<br>• Provide the name of the Institutional Animal Care and Use Committee (IACUC) or other relevant ethics board that reviewed the study protocol, and indicate whether they approved this research or granted a formal waiver of ethical approval<br>• Include an approval number if one was obtained<br>• If the study involved *non-human primates*, add *additional details* about animal welfare and steps taken to ameliorate suffering<br>• If anesthesia, euthanasia, or any kind of animal sacrifice is part of the study, include briefly which substances and/or methods were applied<br><br>**Field Research**<br><br>Include the following details if this study involves the collection of plant, animal, or other materials from a natural setting:<br>• Field permit number<br>• Name of the institution or relevant body that granted permission	
**Data Availability**<br><br>Authors are required to make all data underlying the findings described fully available, without restriction, and from the time of publication. PLOS allows rare exceptions to address legal and ethical concerns. See the PLOS Data Policy and FAQ for detailed information.	Yes - all data are fully available without restriction

A Data Availability Statement describing where the data can be found is required at submission. Your answers to this question constitute the Data Availability Statement and **will be published in the article**, if accepted.<br><br>**Important:** Stating 'data available on request from the author' is not sufficient. If your data are only available upon request, select 'No' for the first question and explain your exceptional situation in the text box.<br><br>Do the authors confirm that all data underlying the findings described in their manuscript are fully available without restriction?	
**Describe where the data may be found in full sentences. If you are copying our sample text, replace any instances of XXX with the appropriate details.**<br><br>• If the data are **held or will be held in a public repository**, include URLs, accession numbers or DOIs. If this information will only be available after acceptance, indicate this by ticking the box below. For example: *All XXX files are available from the XXX database (accession number(s) XXX, XXX.).*<br>• If the data are all contained **within the manuscript and/or Supporting Information files**, enter the following: *All relevant data are within the manuscript and its Supporting Information files.*<br>• If neither of these applies but you are able to provide **details of access elsewhere**, with or without limitations, please do so. For example:<br><br>*Data cannot be shared publicly because of [XXX]. Data are available from the XXX Institutional Data Access / Ethics Committee (contact via XXX) for researchers who meet the criteria for access to confidential data.*<br><br>*The data underlying the results presented in the study are available from (include the name of the third party*	Data underlying this study is readily available in Dryad public data repository with DOI https://doi.org/10.5061/dryad.hhmgqnkfv

and contact information or URL).<br>• This text is appropriate if the data are owned by a third party and authors do not have permission to share the data.<br><br>* typeset	
Additional data availability information:	

Powered by Editorial Manager® and ProduXion Manager® from Aries Systems Corporation

Manuscript Click here to access/download;Manuscript;Manuscript.docx

Prevalence of sexual violence and its associated factors among housemaids attending evening schools in urban settings of Gedeo zone, Southern Ethiopia: A school based cross sectional study

Kalkidan Gezahegn[1], Selamawit Semagn[1], Mohammed Feyisso Shaka[1*]

[1]School of Public Health, College of Health Sciences and Medicine, Dilla University, Dilla Ethiopia

*Correspondence: mohammedshaka2@gmail.com

Abstract

Background: Housemaids are the most vulnerable group to sexual violence due to their working condition, isolation and school arrangements. Despite the ubiquity of sexual violence among the domestic work sector, particularly among housemaids, this area of research continues to be neglected. This study was aimed at examining the prevalence and factors pertaining to sexual violence among housemaids attending night school program in urban setups of Gedeo zone, Southern Ethiopia.

Method: A school based quantitative cross sectional study was conducted among 394 housemaids attending night schools in the urban setups of Gedeo Zone from April to May 2019. After stratifying of students using class grade, SRS technique was used to choose study subjects from each stratum. Quantitative data was collected using face to face interview and qualitative was collected using focus group discussion, in-depth interview and key informant interview. The data was entered and analyzed by SPSS version 20. Binary logistic regression was fitted to determine the association of each independent variable with the dependent variable.

Result: Based on the finding, the prevalence of sexual violence was 60.2%. The odds of experiencing sexual violence through working life-time as housemaid was higher for those who had migrated from rural to urban for work [AOR =1.97: 95 % CI ,(1.07,3.63)], had less than 5 years of experience as housemaid [AOR = 3.10 : 95 % CI , (1.60 , 6.00)], were in the age group of 15-19 [AOR = 3.75 :95 % CI (1.88 ,7.46), ever used alcohol [AOR = 6.77 : 95 % CI,(2.65,17.33)] and whose fathers lacked formal education [AOR= 2.75 :95%CI (1.24,6.08)]. On the other hand, unmarried /housemaids having no regular sexual partner were less likely to face sexual violence [AOR. =0.28: 95% CI, (0.13, 0.57)].

Conclusion: The level of sexual violence was found to be high among housemaids attending night schools in in this study. Housemaids from rural area, those newly starting the work, younger housemaids, and those who were married were more likely to be victims of sexual violence.

Key words: sexual violence; housemaids; evening school; Gedeo zone

Background

Sexual violence is defined as: 'Any sexual act, attempt to obtain a sexual act, unwanted sexual comments or advances, or acts to traffic or otherwise directed against a person's sexuality using coercion, by any person regardless of their relationship to the victim, in any setting, including but not limited to home and work. It can take many forms including a threat of rape, attempted rape, complete rape, sexual harassment and sexual contact with force (1). In any of the forms of sexual violence, minority and marginalized women are the most vulnerable, and generally those who face the greatest obstacles to gain protection and necessary services (2).

Domestic work is an occupation for millions of women worldwide. According to international labor organization (IOL) 2010 estimates, domestic workers represent 4 - 10 % and 1-1.5% of the total workforce in developing and developed countries respectively (3). In Ethiopia, there is a very high prevalence of seasonal and long-term rural-urban migration for domestic work. The main destinations of these migrants are majorly regional and zonal towns, which provide these migrants with domestic services as a housemaid (4). Domestic work is one of the least protected sector under labor law and poor monitoring and implementation of the existing laws puts housemaids in a highly disadvantaged position that in turn exacerbates their vulnerability to sexual abuse and violence (5, 6).

According to evidences from different literatures, the sexual activity of housemaid women also differs from that of the general population. A population based study conducted in urban areas of Ethiopia revealed that, domestic workers were more likely to have had sex before age 15 and to have been coerced into having sex when compared to other young women (9).

Housemaids often comes across different types of sexual abuse ranging from verbal harassment to forced sex by male members of the household, brokers and friends (5, 7-9). Survival becomes primary consideration for the victims because they often have few or no options for other work, and they also lack awareness and knowledge of their options since most come from poor families, are migrants from the rural areas, and have low level of education. As a result, they usually cannot report such offences so that these kinds of violence against house maids remain usually concealed (8, 10, 11).

Most housemaids do not get the opportunity to continue their education on a regular program. Consequently, they are obliged to attend the night shift, which increases their vulnerability to sexual abuse. Many of the studies related to youths conducted in the context of regular secondary schools and higher educational institutions with an emphasis on sexual violence, with very few studies carried out on the most vulnerable group in the evening program
(14-16).

Despite the number of studies revealing the vulnerability of housemaids to sexual abuse and exploitation, there is a substantial lack of data on the magnitude of sexual violence among housemaids in Ethiopia

Methods and materials

Study design and setting

A school based quantitative cross sectional study was conducted in urban settings of Gedeo zone, Southern Ethiopia, from April 1 to May 1, 2019. Gedeo Zone is located at 369 km to the South of Addis Ababa. This study was conducted in **t**he two urban administrative (Dilla and Yirgachefie towns) in the district. The study was conducted in five schools (1 high school & four primary schools) in the two town administrative.

Population and sample size

Female housemaids aged 15 years and above and attending evening schools during the study period in the districts were included. Sample size was determined using single population proportion formula considering the following assumptions: prevalence of sexual violence among housemaids assumed to be 50% due to lack of comparable previous study, 95% certainty and maximum discrepancy of 5 % between the sample size and the underlining population. The adopted sample size formula is:

$$n= (Z\alpha/2)^2 \, p \, (1-p) \, / \, d^2 \qquad (1)$$

Where:

n= sample size

P= prevalence of sexual violence among house maids =50%

d= margin of error 5%

$Z\alpha/2$= critical value at 95% confidence level of certainty (1.96)

Accordingly, the calculated sample size, $n = (1.96)^2 \times 0.5(1 - 0.5) / (0.05)^2 = 384$

Finally 10% of non-response rate was added making the total sample size of this study 422.

Sampling technique

Among the schools having evening program, five schools were randomly selected and census was conducted to identify students who were housemaids. To identify the housemaids, all students attending selected evening schools were asked to fill a form on the type of job they do. Different codes were assigned to different possible types of occupations along with their ages and class role number to identify eligible respondents. Then the total number of housemaid students in each of evening schools were identified with their respective class grades and the sample size was proportionally allocated to each class. In this regard, the distribution of total number of housemaid students in each class were:

- 45 students in grade 9 (no housemaid student was obtained with educational level above grade nine)
- 166 students in grade 7 and 8
- 122 students in grade 5 and 6
- 124 students in grade 3 and 4
- 150 students in grade 1 and 2

With proportional allocation to each size of the total students in each classes, the allotted number of students for each category was:

- 31 students in grade 9
- 116 students in grade 7 and 8
- 85 students in grade 5 and 6
- 86 students in grade 3 and 4
- 104 students in grade 1 and 2

Then simple random sampling technique was used to choose the proportionally allocated study participants from each class. For eligible participants who were absent at the day of data collection, data collectors were revisited the class room two times at different time intervals and for those who were absent after two revisits, the participant was replaced by another in the sampling frame.

Data collection procedure and data quality control

Data was collected with face to face interview by six data collectors using structured questionnaire. The instrument was adapted from previous published literatures and WHO multi-country study on

women’s health (19-21) and it was modified depending on the local situation and the research objective. The tool contains socio demographic characteristics of respondents, family and employers characteristics, behavioral information and sexual history[illegible]he questionnaire was prepared in English, and translated to Amharic to use in the field. The tool was also pre-tested before the actual use in the field. Pre-test was done using 10 house maids attending night schools in Wonago town which is the nearby district to the actual data collection site, with comparable socio-demographic and socio-economic characteristics.

Data collection process was supervised by the investigators and employed supervisors for this purpose. Training was given for data collectors and supervisors to orient them on the objectives and ethical conduct of the study, the nature of the study, the sensitiveness of the issue and how to minimize any bias during interview.

Measurement

Sexual violence experience for each housemaid was defined if a housemaid faces one or more of the following: rape, attempted rape, coerced sex, any sexual harassment at list once in her life-time course as housemaid.

Sexual harassment- when the participant experienced one of the following: unwanted and repeated sexual advances that range from unwelcome comments and improper or offensive verbal jokes, kissing and touching her sensitive body parts.

Problematic substance use or involvement was measured using ASSIST tool. From the measurement, a score of greater than 5 was used for alcoholic beverages (like areke, tela, beer, etc.) and for any other type of substance (shisha, chat, etc.) a score of greater than 2 was considered as problematic substance use or involvement(22)

Data processing and analysis

The quantitative data was checked, coded and entered to Epi-Data version 3.1 and was exported to SPSS (Statistical Package for Social science) version 20 for analysis. In descriptive statistics tables, graphs, mean and frequency was used to present the finding. Binary logistic regression was fitted to identify factors associated with sexual violence. The strength of statistical association between dependent and independent variables was measured at p-value<0.05 by adjusted odds ratio with their corresponding 95% confidence interval.

Ethical Consideration

Ethical clearance was obtained from Institutional Review Board of Dilla University, College of Health Science and Medicine. Written informed consent was obtained from each respondent after detailed explanation of the nature and procedure of the study. Written consent was obtained from the non-minor respondents before conduction of the study. For minors, the assent was obtained from their parents/guardian for those whom their parents/guardian were accessible. But for some minors whom their parents/guardians were not access

.65sible due to different reasons, the IRB was clearly informed about the problem and their assent was waived. The information given by each respondent was kept with strict confidentiality and name was not recorded.

Result

Socio-demographic characteristics of the housemaids

Out of the 422 housemaids that were expected to participate in this study, a total of 394 were interviewed allowing a response rate of 93.4%. It was noted that, the mean age of the study participants was 18 years (SD ±2.5) with the minimum age being 15 years and maximum age being 24 years. Among these, 279 participants (70.8%) were adolescents (aged 15–19 years), whereas 115 (29.2%) were young adults (aged 20–24 years). A majority of the study's participants 316 (80.2%) were neither married nor had a boyfriend. Regarding their educational status, it was found that 264 (67%) participants, i.e. large number of them, were educated only up to the 6th grade or below. In all 277 (70.3%), of the respondents earned between 100–500 birr as monthly salary and an almost equal number of them, i.e., 294 (74.6%) had no family support. As for the family characteristics of the participants, the parents of 135 (34.3%) of them were either divorced or one of the parent had passed away. Educationally, 159 (53.4%) of the respondents' fathers and 251 (63.7%) of the respondents' mothers had no formal education. Out of the total participants, 346 (87.8%) were from families with low income (Table 1).

Table 1: Socio demographic and family characteristic of housemaids attending night schools in urban settings of Gedeo zone southern Ethiopia, May 2019

Variable	Frequency	Percent
Age category	394	100

15-19	279	70.8
20-24	115	29.2
Mean	18± 2.5	
Religion of the respondent	394	100
Orthodox	118	29.9
Protestant	257	65.2
Muslim	12	3.2
Catholic	7	1.8
Marital status of the respondents	394	100
Never married	308	78.1
Regular boyfriend	60	15.22
Married	18	4.5
Divorced/ Widowed	8	2.02
Monthly salary	394	100
≤100	57	14.5
>100-500	277	70.3
>500	60	15.2
Educational level	394	100
First cycle (grade 1-4)	182	46.2
Second cycle (grade 5-8)	188	47.7
High school (grade 9)	24	6.1
Child hood residence	394	100
Rural	288	73.1
Urban	106	26.9
Father educational status	298	100
No formal education	159	53.4
Primary education	98	32.9
Secondary education and above	41	13.8
Mother educational status	332	100
No formal education	251	75.6
Primary education	61	18.4
Secondary education and above	20	6.0
Family income	394	100
Medium	48	12.2
Poor	346	87.8
Family support	394	100
Yes	100	25.4
No	294	74.6

Employment and employer characteristics of the housemaid

A majority of the housemaids, 332 (84.3%), were live-in domestic workers and the remaining 62 (15.7%) of them were live-out workers. The respondents were asked about their residence before

coming to the present place of residence. About 288 (73.1%) of them were from rural areas. Regarding their work experience as housemaids, 286 (72.6%) had worked for more than five years and the mean duration of their work experience was four years with a minimum of one year and a maximum of ten years.

Some information about their employers was also asked of the housemaids who participated in this study. Among the employers, almost all of them were married and 232 (58.9%) had attended higher education *(*Table 2).

Table 2: Employment and employer characteristics of housemaids attending evening schools in urban settings of Gedeo zone Southern Ethiopia, May 2019

Variable	Frequency	**percent**
Agreement type	**394**	**100**
Live-in	332	84.3
Live-out	62	15.7
Duration of residence in the town	394	100
Five years and below	217	55.1
Above five years	177	44.9
Experience as a housemaid	**394**	**100**
Five years and below	286	72.6
Above five years	108	27.4
Employer marital status	**394**	**100**
Married	372	94.4
Single	22	5.6
Occupation of employer	**394**	**100**
Merchant	100	25.4
Government employee	245	62.2
Private	47	11.9
Other	2	0.5
Family size of the employer	**394**	**100**
Less than three	30	7.6
Three to five	166	42.1
Greater than five	198	50.3

Sexual history and behavioral characteristics

Among the total study participants, 103 individuals (26.1%) had a life time history of sexual intercourse, and of those individuals, 55 (53.4%) were sexually active during the past 12 months. The mean age at first sexual intercourse was 17, and for the largest number of the sexually actives, the reason for initiation of sexual activity was forced sex (39 individuals, 37.9%), followed by

personal desire (24 individuals, 23.3%). On the other hand, 53 (13.6%) had ever used alcohol *(Table 3).*

Table 3: sexual history of housemaids attending night schools in urban settings of Gedeo zone southern Ethiopia, May 2019

Variable	Frequency	Percent
Sexual debut	394	100
Yes	103	26.1
No	291	73.9
Age at first sex in years	103	100
10-14	12	11.7
15-19	79	76.7
≥20	12	11.7
Reason for first sex	103	100
Forced	39	37.9
Desire	24	23.3
Marriage	18	17.5
Peer pressure	15	14.6
For money or promising word	7	6.8
Life time regular sexual partner	394	100
None	336	85.3
One	39	9.9
Two and above	19	4.8
Sex in the last 12 months	394	100
Yes	55	14
No	339	86

Life-time substance use and current (within the past 3 months) substance use were assessed using the ASSIST tool. Based on the scoring of the tool, none of the participants fulfilled the criteria to be classified as substance users. The study showed that, 53 (13.3%) of the study participants had ever used alcohol in their life-time, and of that group 45 consumed alcohol once or twice in the past 3 months. Only 8 students (2.0%) had ever chewed chat, and only 2 students (0.5%) had ever smoked shisha. None of the respondents had ever smoked a cigarette.

Prevalence of sexual violence among housemaids

The prevalence of life time sexual violence among the housemaids in this study was 237 (60.2%). The most prevalent type of sexual violence was sexual harassment, reported by 230 (58.4%) of the respondents. The prevalence of completed rape and attempted rape in life-time were 44 (11.2%)

and 75 (19%) respectively (Fig 1). Regarding the perpetuators of the violence, 75 instances (31.6%) were performed by a person unknown by the victim and 54 (22.8%) were perpetrated by the broker or other intermediary person (Table 4).

Table 4: Prevalence of sexual violence among night school housemaid students in urban settings of Geode zone southern Ethiopia, May 2019

**Sexual violence**	**Frequency**	**Percent**
Life-time* sexual violence	**394**	**100**
Yes	237	60.2
No	157	39.8
Perpetuator	237	100
Employer	38	16.0
Employer neighbor	16	6.8
Employer close relative	27	11.4
Broker or other intermediary	54	22.8
Friend	16	6.8
Group	11	4.6
Unknown person	75	31.6
Age of the perpetrator	237	100
Same age	11	4.6
Older than me	81	34.2
Much older than me	146	61.6
Place of occurrence of the event	237	100
Employer home	65	27.3
School compound	38	15.9
Jungle	38	15.9
Hotel	5	2.3
Friends home	10	4.5
Perpetuators home	81	34.1

*Lifetime is a period during the respondents' life as a housemaid

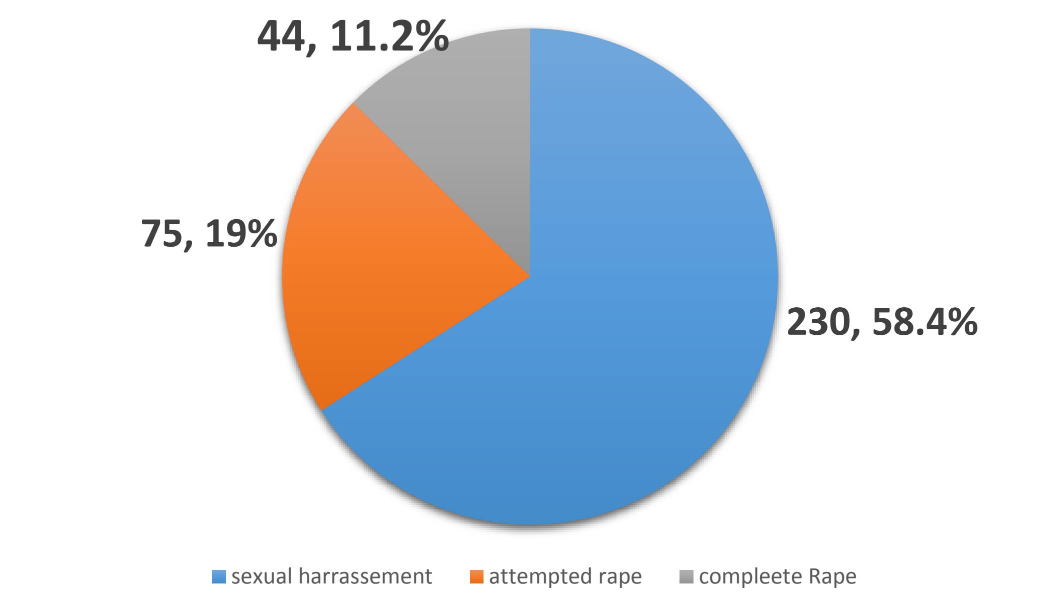

NB. Respondent can report more than one form of sexual violence

Figure 1: Prevalence of different forms of life time sexual violence among housemaids attending night schools in urban setups of Gedeo zone, May 2019

Factors associated with sexual violence among Housemaids

After adjustment for possible confounders on multivariate analysis; age of the housemaid, childhood place of residence, duration of experience as a housemaid, relationship status of the housemaids, ever alcohol use by a housemaid and father's educational level were identified to be significantly associated with life time sexual violence.

The result of multivariable logistic regression analysis revealed that, housemaids aged 15-19 years were about four times [AOR: 3.75, 95% CI (1.88, 7.46] more likely to face sexual violence compared to those greater than or equal to 20 years. The odds of sexual violence was increased by 97% [AOR: 1.97, CI: (1.07, 3.63) for housemaids coming from rural area compared to those from urban. Regarding the year of experience as housemaid, those who worked for less than or equal to five years were three times [AOR: 3.10, CI: (1.60, 6.00)] more likely to be sexually violated compared to those who worked for more than five years.

Among the family characteristics, the odds of life time sexual violence was almost three times [AOR: 2.75, CI: (1.24, 6.08)] for those whose fathers had no formal education compared to those

whose fathers educational level was secondary and above. Among the behavioral characteristics, the odds of life time sexual violence was about seven times [AOR: 6.77, CI: (2.65, 17.33)] higher among housemaids who ever used alcoholic beverages compared to those who never used any kind of alcoholic beverages.

On the other hand, housemaids who were not in a union were less likely to face sexual violence. In this regard, the likelihood of experiencing sexual violence for those who were not in union was decreased by 72% [AOR: 0.28, CI (0.13, 0.57)] compared to those who were in a union (*Table 5)*.

Table 5: Bivariate and multivariable logistic regression analysis result of the factors associated with sexual violence among housemaids attending night schools in urban settings of Gedeo zone Southern Ethiopia May 2019.

**Variable**	**Sexual violence**		**COR(95%CI)**	**AOR (95%CI)**	**P value**
	**Yes**	**No**			
	**Frequency (%)**	**Frequency (%)**			
Age category	237	157			
15- 19years	188(67.4)	91(32.6)	2.78(1.78,4.34)	3.75(1.88,7.46)	<0.001
20-24 years	49(42.6)	66(57.4)	1	1	
Child hood residence	237	157			
Rural	183(63.5)	105(36.5)	1.67(1.07, 2.63)	1.97(1.07,3.63)	0.029
Urban	54(50.9)	52(49.1)	1	1	
Monthly salary (Ethiopian Birr)	237	157			
≤100	45(78.9)	12(21.1)	2.86(1.26, 6.48)	1.88(0.66, 5.39)	0.234
100-500	158(57)	119(43)	1.01(0.57, 1.78)	0. 72(0.32, 1.62)	0.435
>500	34(56.7)	26(43.3)	1	1	
Marital status	237	157			
Not in relationship	179(56.6)	137(43.4)	0.45(0.25 ,0.78)	.28(0.13,0.57)	0.001
In a relation ship	58(74.4)	20(25.6)	1	1	
Experience as a housemaid	237	157			
Five years and below	195(68.2)	91(31.8)	3.36(2.12,5.33)	3.10 (1.60,6.00)	0.001
Above five years	42(38.9)	66(61.1)	1	1	
Duration of stay in the present town	237	157			
Five years and below	146(67.3)	71(32.7)	1.94(1.29,2.92)	0.79(0.41,1.54)	0.499
Above five years	91(51.4)	86(48.6)	1	1	
Alcohol ever use	237	157			

Yes	46(86.8)	7(13.2)	5.16(2.26,11.75)	6.77(2.65,17.33)	<0.001
No	191(56.0)	150(44)	1	1	
Father education	237	157			
No formal education	106(66.7)	53(33.3)	2.31(1.15, 4.64)	2.75(1.24,6.08)	0.012
Primary education	54(55.1)	44(44.9)	1.42(0.68,2.95)	1.38(0.55,3.44)	0.480
Secondary and above	19(46.3)	22(53.7)	1	1	
Family support	237	157			
Yes	51((51.0)	49(49)	1	1	
No	186(63.3)	108(36.7)	1.65(1.04,2.61)	1.56(0.91,2.69)	0.106

Discussion

This study assessed the prevalence of sexual violence and associated phenomena among housemaids attending evening schools in urban settings in the Gedeo Zone. The results indicate that the lifetime prevalence of sexual violence among the housemaids is 60.2% (95% CI: 55.3%–64.5%). Notably, 52.8% of the incidents occurred within the past year. The level of sexual violence among the subjects of this study represents a significant public health concern, and it reflects similar findings in a study conducted in Hosaena, in which 57.75% of the housemaids had faced sexual violence (24). An even greater incidence has been found in studies conducted in larger cities in Ethiopia, including Harar (72%) (7), Addis Ababa (76%) (25), and Mekele (84%) (13). This discrepancy mainly reflects the different contexts of the studies, in which there were larger numbers of the target population in various Ethiopian cities with different cultural and socioeconomic characteristics.

The most common type of sexual violence was sexual harassment, which was reported by 230 (58.4%) of the study's participants, supporting the findings of the study in Hosaena (24). This demonstrates that harassment can occur in numerous ways in diverse social settings. The lifetime prevalence of completed and attempted rape among the study participants was 11.2% and 19.0%, respectively, and the findings of studies from Hawassa (26) and Rwanda (12) reveal comparable levels of rape and attempted rape. Smaller percentages of such incidents were reported in studies from Harar (7), Addis Ababa (21, 25) and Mekelle (13). The extent of diverse types of sexual violence among the study participants in all these contexts and settings indicates a need for broad

action to end violence against women, which remains a considerable obstacle to the achievement of women's and girls' human rights and the 2030 Agenda for Sustainable Development.

Another interesting finding regarding sexual violence among housemaids concerns the condition of their first sexual contact. In this study, 26.1% of the housemaid students had been sexually active, and 37.9% of them were coerced into their first sexual act, revealing the severity of a problem that results in a significant number of housemaids being forced into sex before they intend to do so. This finding is consistent with that of a study conducted in three low-income urban areas in Ethiopia, in which 30.3% of sexually active housemaids described their first sexual experience as occurring under coercive conditions (9).

This study reveals that younger housemaids were more likely to be sexually violated than older ones (above 20 years), a finding consistent with that of the study from Rwanda (12). Younger housemaids are physically as well as psychologically immature and thus more easily manipulated; they may have insufficient experience to enforce clear boundaries for sexual activities and may be less able to object to unwanted advances. Moreover, their lack of maturity may hinder their recognizing of warning signs early, leading perpetrators to more readily target younger housemaids who offer little or no resistance to their sexual predation.

Alcohol use was another factor associated with sexual violence among housemaids. The results show that housemaids who had ever used alcohol were almost seven times more likely to be sexually violated than those who had never drunk alcohol. This finding is in line with the studies conducted among housemaids in Hawassa (26, 27) and Addis Ababa (21). Apparently, consuming alcohol makes it more difficult for housemaids to protect themselves by interpreting and effectively responding to warning signs.

In this study, involvement in a relationship was also significantly associated with lifetime sexual violence. Housemaids who were not in a union or married were 72% less likely to face sexual violence than those who were either married or in a union, suggesting that the main perpetrators of sexual violence were intimate partners. Housemaids with a boyfriend or husband may be less likely to resist attempts at sexual violence for fear that doing so would end the relationship.

The study participants' original area of residence was also significantly associated with lifetime sexual violence; housemaids from rural areas were almost twice as likely to be sexually violated

as those from urban areas. Housemaids from rural areas are less exposed to sexual and reproductive health information because media are not easily accessible in those areas. Additionally, they experience cultural alienation when they migrate to urban areas and may not speak the local language, causing them to face cultural and communication barriers that make them more vulnerable to sexual violence than housemaids from urban areas.

Regarding years of experience as a housemaid, those who had worked for less than five years were three times more likely to be sexually violated than those who had worked for more than five years. This is explained by the fact that housemaids who face any kind of sexual violence are less likely to remain in the sector and more likely to seek different employment to escape an abusive work environment. This conclusion is supported by the finding of a study conducted among out-of-school girls in six Ethiopian regions by the population council (17). Additionally, housemaids who face sexual violence may be fired by female or male employers to conceal the matter and protect the employer's reputation, reducing the likelihood of a housemaid's remaining in the sector. Housemaids who do not face sexual violence are more likely to continue working as a housemaid and may have sufficient life experience to protect themselves from being sexually violated.
Father's level of educational attainment was another factor significantly associated with life time sexual violence among housemaids.. In this regard, housemaids whose father didn't have formal education were more than three times more likely to face sexual violence as compared to those whose fathers educational level was above secondary level. This is possibly due to the fact that those housemaids whose fathers were educated will get information about sexual matters, about their rights, how to protect themselves from being sexually violated and where to go in case of difficulties from their father before entering to domestic work service.

Strength and limitation of the study

This study was conducted to address the marginalized, vulnerable and the most neglected group of population with respect to sexual violence. However, important limitations were admitted in this study. The main limitation of this study was confinement to school attending housemaids which might not reflect the reality among the general population of all housemaids found in the sector. There was also serious limitation of literatures in the area to make more conclusive discussion.

Conclusion and recommendation

In the current study, sexual violence was found to be a major problem among going housemaids. Nearly two-third of the participants have faced at least one form of sexual violence. The life-time completed rape and attempted rape level were also substantially considerable. Younger ages, housemaids who are in union, less experienced housemaids, those residing in rural area prior to the present place of work, and those whose their parent, particularly father was less educated were more likely to experience sexual violence in this study. Thus, pragmatic intervention programs aiming to solve the problem of such vulnerable segment of populations needs to be in place. Government should focus this group particularly, with respect to labor laws and regulations to insure their basic human right. Further evidence generating studies focusing on effective interventions modalities to address this population should be taken in to consideration by researchers.

Funding

Funding for this study was covered by Dilla University NORHED/SENUPH project and the funding organization have no any role in methodology, analysis, interpretation, and write up of the manuscript.

Data Availability

Data underlying this study is readily available in Dryad public data repository with DOI https://doi.org/10.5061/dryad.hhmgqnkfv

Acknowledgement

We would like to acknowledge NORHED project for financial coverage Gedeo zone for their cooperation during conduction of the project. We are also very grateful to study participants and data collectors participated in the study.

REFERENCE

1.World health organization (WHO). Violence against women: intimate partner and sexual violence against women, 2014.

2.Human Rights Watch, Swept under the rug: abuses against domestic workers around the world, Human Rights Watch. 2006. Vol. 18, No. 7(C). 2006.

3.Internationa labour organization (ILO).Decent work for domestic workers. Report IV(1), fourth item on the agenda for (2)International Labour Conference, 99th session 2010.
4.Tadele, F., Pankhurst, A., Bevan, P., & Lavers, T. Migration and rural- urban linkages in Ethiopia and the implications for policy and development practice: Case studies of five rural and two urban sites in Addis Ababa, Amhara, Oromia and SNNP Regions. (Prepared for Irish Aid Ethiopia,2006.
5.Gebre KM. Vulnerability, Legal Protection and Work Conditions of Domestic Workers in Addis Ababa   Human Right, Development and Social Justice 2012
6.Gebremedhin, M. M.. Procrastination in Recognizing the Rights of Domestic Workers in Ethiopia. Mizan Law Review, 2016 10(1), 38. https://doi.org/10.4314/mlr.v10i1.2.
7.Mustefa A. Sexual Violence against Women Domestic Worker Clients at Harar Higher Sexual and Reproductive Health Model Clinic of Family Guidance Association of Ethiopia, Eastern Ethiopia MSW Dissertation Research Project Report(MSWP - 001) , August 2014.
8.Biadegilegne, E.. Condition of work for adult female live-in paid domestic workers in Addis Ababa, Ethiopia,. 2011Kimmage DSC, Development Studies Centre.
9.Erulkar A and Ferede A. Social exclusion and early or unwanted sexual initiation among poor urban females in Ethiopia. International Perspec¬tives on Sexual and Reproductive Health,2009. 35(4): 186–193.).
10.Tobarak K and Rashid S.Violence against Women: Nature, Causes and Dimensions in Contemporary Bangladesh, Bangladesh e-Journal of Sociology.2013 10(1)79 ,,.
11.Aynalem s, Causes and Consequences of Sexual Abuse and Resilience Factors in Housemaids Working in Addis Ababa: A Qualitative Inquiry . The Ethiopian Journal of Social Sciences 2015 .Volume 1, Number 1,.
12.CLADHO and IDAY-International .Domestic workers' baseline survey focusing on child domestic workers and employers in Rwanda Kigali, July 2015
13. Gebremariam L, Tukue D, Desaleg S et al. Prevalence of Sexual Violence and its Associated Factors among Female Night Time Students of Junior and Secondary Schools in Mekelle City, Northern Ethiopia, Journal of Medical Science and Technology (RRJoMST). 2015; 4(2):   6–20p.
14.Asfaw M .Sexual Violence and its Consequence among Female Night School Students In Hawassa town, Southern Ethiopia: A cross-sectional study. 2010.

15.Belete Y M. challenges and opportunities of female domestic workers in accesing education a qualitative study from Bahir Dar city administration ,Amhara region ,Ethiopia April 2014.
16. Altinyelken .HK & Mat ,LM Sexual violence, schooling and silence: teacher narratives from a secondary school in Ethiopia, Compare: A Journal of Comparative and International Education .2018.48:4, 648-664, DOI: 10.1080/03057925.2017.1332517.
17.Erulkar, A., G. Medhin, and L. Negeri. .The Journey of Out-of-School Girls in Ethiopia: Examining Migration, Livelihoods, and HIV.2017 Addis Ababa: Population Council. .
18.Gedeo zone health office annual plan & achievement, 2011 E.C.
19.Statistical surveys Department of Economic and Social Affairs Statistics Division ,Guidelines for Producing Statistics on Violence against Women— United NationsNew York, 2014.
20.Garcia-Moreno C et al. WHO multi-country study on women's health and domestic violence against women: initial results on prevalence, health outcomes and women's responses. Geneva, World Health Organization, 2005.
21.Getachew M .Prevalence and determinants of sexual violence among female housemaids in selected junior secondary night school:cross sectional study Addis Ababa, Ethiopia ,2015.
22.DRUG and ALCHOL service south AustraliaASSIST for young people aged 15-17 draft clinical form ,2011.
23.Dilla town first stage court house rape victims of 2011 Ethiopia calander individual folders
24.Tagesse L ,Dubey R, Forms of Violence against Female Domestic Workers :The Case of Hossana Town, Hadiyya Zone SNNPR, Ethiopia Imperial Journal of Interdisciplinary Research (IJIR) .2017 Vol-3, Issue-8, ISSN: 2454-1362,.
25.Getachew Y. Addis Ababa University of Graduate studies Cross sectional assessment of violence against female domestic workers in gulele sub-city for local level intervention 2006.
26.Esatu F Sexual violence among house maids and its adverse reproductive health outcomes in Hawassa, Ethiopia an open access journal Volume 8, Issue 6 (Suppl).2017. ISSN: 2155-6113.
27. Yohannes K, Abebe L, Kisi T, Demeke W, Yimer S, Feyisso M, et al. The prevalence and predictors of domestic violence among pregnant women in Southeast Oromia, Ethiopia. Reprod Health 2019;16. https://doi.org/10.1186/s12978-019-0694-9.

Prevalence of sexual violence and its associated factors among housemaids attending evening schools in urban setups of Gedeo zone, Southern Ethiopia: A school based cross sectional study

Kalkidan Gezahegn[1], Selamawit Semagn[1], Mohammed Feyisso Shaka[1*]

[1]School of Public Health, College of Health Sciences and Medicine, Dilla University, Dilla Ethiopia

*Correspondence: ~~mamfys8@gmail.com~~mohammedshaka2@gmail.com

Abstract

Formatted: Font: 18 pt

Background: Housemaids are the most vulnerable group to sexual violence due to their working condition, isolation and ~~their~~ school arrangements. Despite ~~commonness of~~the ubiquity of sexual violence among the domestic work sector, particularly among housemaids, this area of research continues to be neglected.~~it is a neglected area of research.~~ This study was aimed ~~to assess~~at examining the prevalence and factors ~~related~~ pertaining to sexual violence among housemaids attending night school program in urban setups of Gedeo zone, Southern Ethiopia.

Method: A school based quantitative cross sectional study was ~~done~~ conducted among 394 housemaids attending night schools in the urban setups of Gedeo Zone from April to May 2019. After ~~stratification~~ stratifying of students using class grade, SRS technique was used to ~~select~~ choose study subjects from each stratuma~~a~~. Quantitative data was collected using face to face interview and qualitative was collected using focus group discussion, in-depth interview and key informant interview. The data was entered and analyzed by ~~using~~ SPSS version 20. Binary logistic regression was fitted to determine the association of each independent variable with the dependent variable.

Result: ~~From~~ Based on the finding, the prevalence of sexual violence was 60.2%. The odds of experiencing sexual violence through working life time as housemaid was higher for those who had migrated from rural to urban for work [AOR =1.97: 95 % CI ,(1.07,3.63)], ~~have~~ had less than 5 years of experience as housemaid [AOR = 3.10 : 95 % CI , (1.60 , 6.00)], were in the age group of 15-19 [AOR = 3.75 :95 % CI (1.88 ,7.46), ever used alcohol [AOR = 6.77 : 95 % CI,(2.65,17.33)] and ~~whom fathers did not have~~whose fathers lacked formal education [AOR= 2.75 :95%CI (1.24,6.08)]. On the other hand, unmarried /housemaids having no regular sexual partner were ~~found to be~~ less likely to ~~have~~ face sexual violence [AOR. =0.28: 95% CI, (0.13, 0.57)].

Conclusion: The level of sexual violence was found to be high among housemaids attending night schools in in this study. Housemaids from rural area, those newly starting the work, younger housemaids, and those who were married were more likely to be victims ~~for~~ of sexual violence.

Key words: sexual violence; housemaids; evening school; Gedeo zone

Background

Formatted: Font: 18 pt

Sexual violence is -defined as: 'Any sexual act, attempt to obtain a sexual act, unwanted sexual comments or advances, or acts to traffic or otherwise directed against a person's sexuality using coercion, by any person regardless of their relationship to the victim, in any setting, including but not limited to home and work. It can take many forms including a threat of rape, attempted rape, complete rape, sexual harassment and sexual contact with force (1). In any of the forms -of sexual violence, minority and marginalized women are the most vulnerable, and generally those who face the greatest obstacles to gain protection and necessary services (2).

Domestic work is an occupation for millions of women worldwide. According to international labor organization (IOL) -2010 estimates-, domestic workers represent 4 - 10 % and 1-1.5% of the total workforce in developing and developed countries respectively (3). In Ethiopia, there is a very high prevalence of seasonal and long-term rural-urban migration for domestic work. The main destinations of these migrants are majorly regional and zonal towns, which provide these migrants with domestic services as a housemaid (4). Domestic work is one of the least protected sector under labor law and poor monitoring and implementation of the existing laws puts housemaids in a highly disadvantaged position that in turn exacerbates their vulnerability to sexual abuse and violence (5, 6).

According to Eevidences from different literatures, also shows that, the sexual activity of housemaid women also differs from that of the general population. A population based study which was conducted on in urban areas of Ethiopia reveaiiled that, compared to other young women, domestic workers were more likely to have had sex before age 15 and to have been coerced into having sex when compared to other young women (9).

Housemaids often comes across different types of sexual abuse ranging from verbal harassment to forced sex by male members of the household, brokers and friends (5, 7-9). Survival becomes primary consideration for the victims because they often have few or no options for other work, and they also lack awareness and knowledge of their options since most come from poor families, are migrants from the rural areas, and have low level of education. As a result, they usually cannot report such offences so that these kinds of violence against house maids remain usually concealed (8, 10, 11).

Most housemaids do not get the opportunity to continue their education on a regular program. Consequently, they are obliged to attend the night shift, which increases their vulnerability to sexual abuse. Many of the studies related to youths conducted in the context of regular secondary schools and higher educational institutions with an emphasis on sexual violence, with very few studies carried out on the most vulnerable group in the evening program

Formatted: Font: Font color: Black

Formatted: Default, Line spacing: 1.5 lines

Formatted: Font: Bold, Font color: Black

~~Most of housemaids do not get the opportunity to continue their education on regular base so that they are forced to attend the night shift and that will increase their vulnerability to sexual abuse. But Many of the studies related to youth are conducted in the context of regular secondary schools and higher educational institutions with emphasis on sexual violence, with very few studies carried out on the most vulnerable group in the evening program~~(14-16).

Despite the number of studies revealing the vulnerability of housemaids to sexual abuse and exploitation, there is a substantial lack of data on the magnitude of sexual violence among housemaids in Ethiopia

Formatted: Font: Font color: Black

Formatted: Default, Line spacing: 1.5 lines

Formatted: Font: Bold, Font color: Black

~~Despite the number of studies revealing vulnerability of this group for sexual abuse and exploitation, there is a serious lack of data on the magnitude of sexual violence among house maids in Ethiopia~~.

Methods and materials

Formatted: Font: 18 pt

Study design and setting

Formatted: Font: 16 pt

A school based quantitative cross sectional study was conducted in urban setups of Gedeo zone, Southern Ethiopia from April 1 to May 1, 2019. Gedeo Zone is located at 369 km to the South of Addis Ababa. This study was conducted in the two urban administrative (Dilla and Yirgachefie towns) in the district. The study was conducted in five schools (1 high school & four primary schools) in the two town administrative.

Population and sample size

Formatted: Font: 16 pt

Female housemaids aged 15 years and above and attending evening schools during the study period in the districts were included. Sample size was determined using single population proportion formula considering the following assumptions: prevalence of sexual violence among housemaids assumed to be 50% due to lack of comparable previous study, 95% certainty and

maximum discrepancy of 5 % between the sample size and the underlining population. The adopted sample size formula is:

$$n = (Z\alpha/2)^2 \, p\,(1-p) / d^2 \qquad (1)$$

Where:

n= sample size

P= prevalence of sexual violence among house maids =50%

d= margin of error 5%

Zα/2= critical value at 95% confidence level of certainty (1.96)

Accordingly the calculated sample size, $n = (1.96)^2 \times 0.5(1 - 0.5) / (0.05)^2 = 384$

Finally 10% of non-response rate was added making the total sample size of this study 422.

Sampling technique

Among the schools which have evening program, five schools were randomly selected and census was conducted to identify students who are housemaids. To identify the housemaids, all students attending selected evening schools were asked to fill a form on the type of job they do with different codes assigned to different possible types of occupations along with their ages and class role number to identify eligible respondents. Then the total number of housemaid students in each of evening schools were identified with their respective class grades and the sample size was proportionally allocated to each class. In this regard, the distribution of total number of housemaid students in each class were:

- 45 students in grade 9
- 166 students in grade 7 and 8
- 122 students in grade 5 and 6
- 124 students in grade 3 and 4
- 150 students in grade 1 and 2

With proportional allocation to each size of the total students in each classes, the allotted number of students for each category was:

- 31 students in grade 9 (no housemaid student was obtained with educational level above grade nine)
- 116 students in grade 7 and 8
- 85 students in grade 5 and 6

Formatted: Tab stops: 6.5", Right

Formatted: Font: 16 pt

Formatted: Font: (Default) Times New Roman, 12 pt

Formatted: List Paragraph, Bulleted + Level: 1 + Aligned at: 0.3" + Indent at: 0.55"

Formatted: Font: (Default) Times New Roman, 12 pt

- 86 students in grade 3 and 4
- 104 students in grade 1 and 2

Then simple random sampling technique was used to choose the proportionally allocated study participants from each class. For eligible participants who were absent at the day of data collection, data collectors were revisited the class room two times at different time intervals and for those who were absent after two revisits, the participant was replaced by another in the sampling frame.

Data collection procedure and data quality control

Data was collected with face to face interview by six data collectors using structured questionnaire. The instrument was adapted from previous published literatures and WHO multi-country study on women's health (19-21) and it was modified depending on the local situation and the research objective. The questionnaire was prepared in English, and translated to Amharic to use in the field. The tool was also pre-tested before the actual use in the field. Pre-test was done using 10 house maids attending night schools in Wonago town which is the nearby district to the actual data collection site, with comparable socio-demographic and socio-economic characteristics.

Data collection process was supervised by the investigators and employed supervisors for this purpose. Training was given for data collectors and supervisors to orient them on the objectives and ethical conduct of the study, the nature of the study, the sensitiveness of the issue and how to minimize any bias during interview.

Measurement

Sexual violence experience for each housemaid was defined if a housemaid faces one or more of the following: rape, attempted rape, coerced sex, any sexual harassment at list once in her lifetime course as housemaid.

Sexual harassment- when the participant experienced one of the following: unwanted and repeated sexual advances that range from unwelcome comments and improper or offensive verbal jokes, kissing and touching her sensitive body parts during her working life time as a housemaid

Problematic substance use or involvement was measured using ASSIST tool. From the measurement, a score of greater than 5 was used for alcoholic beverages (like areke, tela, beer, etc.) and for any other type of substance (shisha, chat, etc.) a score of greater than 2 was considered as problematic substance use or involvement(22)

Formatted: List Paragraph, Bulleted + Level: 1 + Aligned at: 0.3" + Indent at: 0.55"

Formatted: Font: (Default) Times New Roman, 12 pt

Formatted: Font: (Default) Times New Roman, 12 pt

Formatted: Font: (Default) Times New Roman

Formatted: Font: (Default) Times New Roman, 12 pt

Formatted: Font: 16 pt, Font color: Auto

Formatted: Font: 16 pt, Bold

Formatted: Heading 2, Space After: 0 pt

Formatted: Font: 16 pt

Formatted: Font color: Auto

Data processing and analysis

Formatted: Font: 16 pt

The quantitative data was checked, coded and entered to Epi-Data version 3.1 and was exported to SPSS (Statistical Package for Social science) version 20 for analysis. In descriptive statistics tables, graphs, mean and frequency was used to present the finding. Binary logistic regression was fitted to identify factors associated with sexual violence. The strength of statistical association between dependent and independent variables was measured at p-value<0.05 by adjusted odds ratio with their corresponding 95% confidence interval.

Ethical Consideration

Formatted: Font: 16 pt

Ethical clearance was obtained from Institutional Review Board of Dilla University, College of Health Science and Medicine. Written informed consent was obtained from each respondent after detailed explanation of the nature and procedure of the study. ~~The information given by each respondent was kept with strict confidentiality and name was not recorded.~~Written consent was obtained from the non-minor respondents before conduction of the study. For minors, the consent was obtained from their parents/guardian for those whom their parents/guardian were accessible. But for some minors whom their parents/guardians were not accessible due to different reasons, the IRB was clearly informed about the problem and their consent was waived. The information given by each respondent was kept with strict confidentiality and name was not recorded.

Result

Formatted: Font: 18 pt

Socio-demographic characteristics of the housemaids

Formatted: Font: 16 pt

Out of the 422 housemaids that were expected to participate in this study, a total of 394 were interviewed ~~making~~ allowing ~~the~~ a response rate of 93.4%. ~~From the result~~It was noted that, the mean age of the study participants was 18 years (SD ±2.5) with the minimum age ~~of~~ being 15 years and maximum age ~~of~~ being 24 years. Among these, 279 ~~(70.8%) of the~~ participants (70.8%) were adolescents (aged 15–~~-~~19 years ~~old~~), whereas 115 (29.2%) were young adults (aged 20–~~-~~24 years). A ~~M~~majority of the study's participants 316 (80.2%) were neither married nor had a boyfriend ~~currently~~. ~~With regard to~~Regarding their educational status, it was found that 264 (67%) participants, i.e. large number of them ~~respondents, 264 (67%)~~, were educated only up to the ~~less than~~ 6th grade or below. ~~Majority,~~In all 277 (70.3%), of the respondents earned between 100–~~-~~

Formatted: Font: Times New Roman

500 birr as monthly salary and ~~two hundred ninety four~~an almost equal number of them, i.e., 294 (74.6%) ~~of them have~~had no family support.

~~Regarding~~As for the family characteristics of the participants, ~~for~~ the parents of 135 (34.3%) of them ~~respondents, their parents~~ were either divorced or one of the parent ~~is not alive~~had passed away. Educationally, 159 (53.4%) of the respondents' fathers and 251 (63.7%) of the respondents' mothers ~~have~~ had no formal education. Out of the total participants, 346 (87.8%) ~~of them are~~were from families with ~~poor~~ low income (Table 1).

Table ~~2~~1: Socio demographic and family characteristic of housemaids attending night schools in urban ~~setups~~ settings of Gedeo zone southern Ethiopia, ~~April~~ May 2019

**Variable**	**Frequency**	**Percent**
Age category	394	100
15-19	279	70.8
20-24	115	29.2
Mean	18± 2.5	
Religion of the respondent	394	100
Orthodox	118	29.9
Protestant	257	65.2
Muslim	12	3.2
Catholic	7	1.8
Marital status of the respondents	394	100
Never married	308	78.1
Regular boyfriend	60	15.22
Married	18	4.5
Divorced/ Widowed	8	2.02
Monthly salary	394	100
≤100	57	14.5
>100-500	277	70.3
>500	60	15.2
Educational level	~~194~~ 394	100
First cycle (grade 1-4)~~1-6~~	~~264~~182	~~67.0~~46.2
~~7-8~~Second cycle (grade 5-8)	~~106~~188	~~26.9~~47.7
~~>=9~~High school (grade 9)	24	6.1
Child hood residence	394	100
Rural	288	73.1
Urban	106	26.9
~~Family situation~~	~~394~~	~~100~~
~~Living together~~	~~226~~	~~57.4~~
~~Divorced~~	~~42~~	~~10.7~~
~~Only One parent alive~~	~~93~~	~~23.6~~

Formatted Table

Formatted Table

Formatted Table

~~Both not alive~~	~~33~~	~~8.4~~
Father educational status	298	100
No formal education	159	53.4
Primary education	98	32.9
Secondary education and above	41	13.8
Mother educational status	332	100
No formal education	251	75.6
Primary education	61	18.4
Secondary education and above	20	6.0
Family income	394	100
Medium	48	12.2
Poor	346	87.8
Family support	394	100
Yes	100	25.4
No	294	74.6

Employment and employer characteristics of the housemaid

A ~~M~~majority of the housemaids, 332 (84.3%), were live-in domestic workers and the ~~rest~~ remaining ~~sixty two~~62 (15.7%) of them were live-out workers. The respondents were asked about their residence before coming to the ~~current~~ present place of residence. ~~place and two hundred eighty eight~~About 288 (73.1%) of them were from rural areas. Regarding their work experience as housemaids, 286 (72.6%) ~~of them~~had worked for more than ~~5~~ five years and the mean duration of their work experience ~~as a house maid~~ was four years with a minimum of one year and a maximum of ten years.

~~Possible~~ Some information ~~of their~~about their employers was also asked ~~from~~ of the housemaids who participated in this study. Among the employers, almost all of them were ~~currently~~ married and 232 (58.9%) had attended higher education (Table ~~3~~2).

Table 2~~3~~: Employment and employer characteristics of housemaids attending evening schools in urban ~~setups~~ settings of Gedeo zone Southern Ethiopia, May 2019

Variable	Frequency	**percent**
Agreement type	**394**	**100**
Live-in	332	84.3
Live-out	62	15.7
Duration of residence in the town	**394**	**100**
~~≤5~~ Five years **and below**	217	55.1
~~>5 years~~Above five years	177	44.9

Formatted Table

Formatted Table

Formatted: Font: 16 pt

Formatted Table

Formatted: Font: Bold

Formatted Table

Experience as a housemaid	**394**	**100**
Five years and below~~≤5 years~~	286	72.6
Above five years ~~>5 years~~	108	27.4
**Employer marital status**	**394**	**100**
**Married**	372	94.4
Single	22	5.6
**Occupation of employer**	**394**	**100**
**Merchant**	100	25.4
**Government employee**	245	62.2
**Private**	47	11.9
**Other**	2	0.5
**Family size of the employer**	**394**	**100**
~~1–2~~**Less than three**	30	7.6
~~3-5~~**Three to five**	166	42.1
~~>5~~**Greater than five**	198	50.3
~~**Perceived employers wealth**~~	~~394~~	~~100~~
~~**Better off**~~	~~106~~	~~26.9~~
~~**Medium**~~	~~285~~	~~72.3~~
~~**Poor**~~	~~3~~	~~0.8~~

Sexual ~~and~~ history and behavioral characteristics

Among the total study participants, 103 individuals (26.1%) ~~of them~~ had a life time history of sexual intercourse, and ~~among them~~of those individuals, 55 (53.4%) were sexually active during the p~~l~~ast 12 months. The mean age at first sexual intercourse was 17, and for the largest~~r~~ number of the sexually actives, the reason for initiation of sexual activity was forced sex (39 individuals, ~~(~~37.9%), followed by personal desire (24 individuals, ~~(~~23.3%). On the other hand, 53 (13.6%) had ever used alcohol *(Table 34)*.

Table 34: sexual history of housemaids attending night schools in urban ~~setups~~ settings of Gedeo zone southern Ethiopia, May 2019

Variable	Frequency	Percent
Sexual debut	394	100
Yes	103	26.1
No	291	73.9
Age ~~of~~ at first sex	103	100
10-14	12	11.7
15-19	79	76.7
≥~~>=~~20	12	11.7
Reason for first sex	103	100
Forced	39	37.9

Formatted Table
Formatted: Font: 12 pt
Formatted: Font: 12 pt, Not Bold
Formatted: Font: Bold
Formatted: Font: 12 pt
Formatted: Font: 12 pt
Formatted: Font: 12 pt
Formatted: Font: 12 pt
Formatted: Font: Not Bold
Formatted: Font: 12 pt
Formatted: Font: 12 pt, Bold
Formatted: Font: 12 pt
Formatted: Font: 12 pt
Formatted: Font: 12 pt
Formatted: Font: 12 pt, Not Bold
Formatted: Font: 12 pt
Formatted: Font: 12 pt
Formatted: Font: 12 pt
Formatted: Font: 12 pt
Formatted: Font: 12 pt
Formatted: Font: 12 pt, Not Bold
Formatted: Font: 12 pt
Formatted: Font: 12 pt
Formatted: Font: 12 pt
Formatted: Font: 12 pt, Not Bold
Formatted: Font: 12 pt
Formatted: Font: 12 pt
Formatted: Font: 12 pt
Formatted: Font: 12 pt
Formatted: Font: 12 pt
Formatted: Font color: Auto

Desire	24	23.3
Marriage	18	17.5
Peer pressure	15	14.6
For money or promising word	7	6.8
Life time regular sexual partner	394	100
None	336	85.3
One	39	9.9
Two and above	19	4.8
Sex in the last 12 months	394	100
Yes	55	14
No	339	86

Life-time substance use and current (within the past 3 months) substance use were assessed using the ASSIST tool. Based on the scoring of the tool, none of the participants fulfilled the criteria to be classified as substance users. The study showed that, 53 (13.3%) of the study participants had ever used alcohol in their life-time, and of that group 45 consumed alcohol once or twice in the past 3 months. Only 8 students (2.0%) had ever chewed chat, and only 2 students (0.5%) had ever smoked shisha. None of the respondents had ever smoked a cigarette.

Prevalence of sexual violence among housemaids

The prevalence of life time sexual violence among the housemaids in this study was 237 (60.2%). The most prevalent type of sexual violence was sexual harassment, reported by 230 (58.4%) of the respondents. The prevalence of completed rape and attempted rape in life--time ~~was~~ were 44 (11.2%) and 75 (19%) respectively (Fig 1). Regarding the perpetuators of the violence, 75 instances (31.6%) ~~was~~ were performed by a person unknown by the victim and 54 (22.8%) ~~was~~ were perpetrated by the broker or other intermediary person (Table 4~~5~~).

Table ~~5~~4: Prevalence of sexual violence among night school housemaid students in urban setup of Geode zone southern Ethiopia, May 2019

Sexual violence	Frequency	Percent
Lifetime* sexual violence	**394**	**100**
Yes	237	60.2
No	157	39.8
Perpetuator	237	100
Employer	38	16.0
Employer neighbor	16	6.8
Employer close relative	27	11.4
Broker or other intermediary	54	22.8

Formatted: Font: Not Italic
Formatted: Font: Not Italic
Formatted: Font: Not Italic
Formatted: Font: Not Italic
Formatted: Font: Not Italic
Formatted: Font: Not Italic
Formatted: Font: Not Italic
Formatted: Font: Not Italic
Formatted: Font: Not Italic
Formatted: Font: Not Italic
Formatted: Font: Not Italic
Formatted: Font: Not Italic
Formatted: Font: Not Italic
Formatted: Font: Not Italic
Formatted: Font: Not Italic
Formatted: Font: Not Italic
Formatted: Font: Not Italic
Formatted: Font: Not Italic
Formatted: Font: 16 pt

Friend	16	6.8
Group	11	4.6
Unknown person	75	31.6
Age of the perpetrator	237	100
Same age	11	4.6
Older than me	81	34.2
Much older than me	146	61.6
Place of occurrence of the event	237	100
Employer home	65	27.3
School compound	38	15.9
Jungle	38	15.9
Hotel	5	2.3
Friends home	10	4.5
Perpetuators home	81	34.1

*Lifetime is a period during the respondents' life as a housemaid

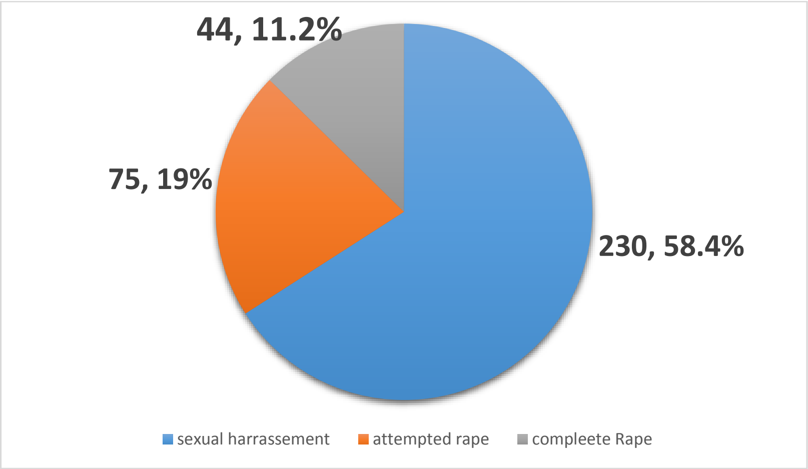

NB. Respondent ~~could~~ can report more than one form of sexual violence

Figure 1: Prevalence of different forms of life time sexual violence among housemaids attending night schools in urban setups of Gedeo zone, May 2019

Factors associated with sexual violence among Housemaids

Formatted: Font: 16 pt

After adjustment for possible confounders on multivariate analysis; age of the housemaid, childhood place of residence, duration of experience as a housemaid, relationship status of the

housemaids, ever alcohol use by a housemaid and father's educational level were identified to be significantly associated with life time sexual violence.

The result of multivariable logistic regression analysis revealed that, housemaids aged 15-19 years ~~of age~~ were about four times [AOR: 3.75, 95% CI (1.88, 7.46] more likely to face sexual violence ~~as~~ compared to those greater than or equal to 20 years. The odds of sexual violence was increased by 97% [AOR:~~=~~ 1.97,~~:~~ CI: (1.07, 3.63) for ~~those~~ housemaids coming from rural area compared to those from urban. Regarding the year of experience as housemaid, those who worked for ~~≤5~~less than or equal to five years ~~as housemaids~~ were three times [AOR: 3.10, CI: (1.60, 6.00)] more likely to be sexually violated ~~as~~ compared to those who worked for more than five years.

Among the family characteristics, the odds of life time sexual violence was almost three times [AOR: ~~R=~~2.75,~~:~~ CI~~,~~: (1.24, 6.08)] for those whose fathers had no formal education ~~as~~ compared to those whose fathers educational level was secondary and above. Among the behavioral characteristics, the odds of life time sexual violence was about seven times [AOR: 6.77, CI: (2.65, 17.33)] higher among housemaids who ever used alcoholic beverages compared to those who never used any kind of alcoholic beverages.

On the other hand, housemaids who were not in a union were ~~found to be~~ less likely to face sexual violence. In this regard, the likelihood of experiencing sexual violence for ~~housemaids~~ those who were not in union was decreased by 72% [AOR: 0.28~~;~~, CI (0.13, 0.57)] compared to those who were in a union (*Table 5~~6~~*).

Table 5~~6~~: Bivariate and m~~M~~ultivaria~~t~~ble logistic regression analysis ~~between~~ result of the factors associated with sexual violence ~~dependent and independent variable~~ among housemaids attending night schools in urban setups of Gedeo zone S~~s~~outhern Ethiopia May 2019.~~(n=394)~~

**Variable**	**Sexual violence**		**COR(95%CI)**	**AOR (95%CI)**	**P valu**
	**Yes**	**No**			
	**Frequency (%)**	**Frequency (%)**			
Age category	237	157			
15- 19years	188(67.4)	91(32.6)	2.78(1.78,4.34)	3.75(1.88,7.46)	<0.001(
20-24 years	49(42.6)	66(57.4)	1	1	
Child hood residence	237	157			
Rural	183(63.5)	105(36.5)	1.67(1.07, 2.63)	1.97(1.07,3.63)	0.029

Formatted: Font: 12 pt
Formatted: Font: 12 pt
Formatted: Font: 12 pt
Formatted Table
Formatted: Font: 12 pt
Formatted: Font: 12 pt
Formatted: Font: 12 pt
Formatted: Font: 12 pt
Formatted: Font: 12 pt

Urban	54(50.9)	52(49.1)	1	1	
Monthly salary (Ethiopian Birr)	237	157			
≤100	45(78.9)	12(21.1)	2.86(1.26, 6.48)	1.88(0.66, 5.39)	0.234
100-500	158(57)	119(43)	1.01(0.57, 1.78)	0. 72(0.32, 1.62)	0.435
>500	34(56.7)	26(43.3)	1	1	
Marital status	237	157			
Not in relationship	179(56.6)	137(43.4)	0.45(0.25 ,0.78)	.28(0.13,0.57)	0.001
In a relation ship	58(74.4)	20(25.6)	1	1	
Experience as a housemaid	237	157			
Five years and below~~≤5~~	195(68.2)	91(31.8)	3.36(2.12,5.33)	3.10 (1.60,6.00)	0.001
Above five years ~~>5~~	42(38.9)	66(61.1)	1	1	
Duration of stay in the present town	237	157			
Five years and below~~≤5~~	146(67.3)	71(32.7)	1.94(1.29,2.92)	0.79(0.41,1.54)	0.499
Above five years ~~>5~~	91(51.4)	86(48.6)	1	1	
Alcohol ever use	237	157			
Yes	46(86.8)	7(13.2)	5.16(2.26,11.75)	6.77(2.65,17.33)	<0.001
No	191(56.0)	150(44)	1	1	
Father education	237	157			
No formal education	106(66.7)	53(33.3)	2.31(1.15, 4.64)	2.75(1.24,6.08)	0.012
Primary education	54(55.1)	44(44.9)	1.42(0.68,2.95)	1.38(0.55,3.44)	0.480
Secondary and above	19(46.3)	22(53.7)	1	1	
Family support	237	157			
Yes	51((51.0)	49(49)	1	1	
No	186(63.3)	108(36.7)	1.65(1.04,2.61)	1.56(0.91,2.69)	0.106

Discussion

~~Prevalence of sexual violence and associated factors among housemaid students attending evening schools in urban setups of Gedeo Zone was assessed in this study. From the result, the life time prevalence of sexual violence among housemaids in this study was 60.2% with 95% CI of 55.3% to 64.5%. Consequently, 52.8% of this was happened to the victims within the last one year.~~

~~The prevalence sexual violence among housemaids in this study is comparable to a finding from the study conducted in Hossana town where 57.75% of housemaids had faced sexual violence (24). but slightly lower than the findings of studies conducted in Harar city (72%)(7), Addis Ababa~~

Formatted: Font: 12 pt
Formatted: Font: 12 pt
Formatted: Font: 12 pt
Formatted Table
Formatted: Font: 12 pt
Formatted: Font: 12 pt
Formatted: Font: 12 pt
Formatted: Font: 12 pt
Formatted: Font: 12 pt
Formatted: Font: 12 pt
Formatted: Justified, Tab stops: Not at 3.18"
Formatted: Font: (Default) Times New Roman, 12 pt, Not Bold
Formatted: Justified, Tab stops: Not at 2.93"
Formatted: Font: 12 pt
Formatted: Font: (Default) Times New Roman, 12 pt, Not Bold
Formatted: Justified, Tab stops: Not at 2.93"
Formatted: Font: 12 pt
Formatted: Font: 12 pt
Formatted: Justified, Tab stops: Not at 2.93"
Formatted: Font: (Default) Times New Roman, 12 pt, Not Bold
Formatted: Justified, Tab stops: Not at 2.93"
Formatted: Font: 12 pt
Formatted: Font: (Default) Times New Roman, 12 pt, Not Bold
Formatted: Justified, Tab stops: Not at 2.93"
Formatted: Font: 12 pt
Formatted: Font: 12 pt
Formatted: Font: 12 pt
Formatted: Font: 12 pt
Formatted: Font: 12 pt
Formatted: Font: 12 pt
Formatted Table
Formatted: Font: 12 pt
Formatted: Font: 12 pt
Formatted: Font: 12 pt
Formatted: Font: 12 pt
Formatted: Font: 12 pt
Formatted: Font: 18 pt
Formatted: Space After: 6 pt

(76%) (25), and Mekele (84%) (13). This might be due to the difference in the context of study where the mentioned cities are among the mega cities in the country with larger number of target population from different parts of Ethiopia with different cultural, and socio-economic backgrounds.

Regarding the types of sexual violence the most common one was sexual harassment reported by 230 (58.4%) of the study participants. This finding is also comparable to the study in hosanna town(24). In this study life time prevalence of completed rape and attempted rape among the study participants was 11.2% and 19 % respectively. This finding in line with the study from Hawassa city (26) and Rwanda(12), but lower than the study from Harar city (7) , Addis Ababa (21, 25) and Mekelle city(13).

Another interesting finding with respect to the sexual violence among housemaids in this study is the condition of their first sexual contact. In this study 26.1% of housemaid students were sexually active and 37.9% of them had their firs sex due to coercion, revealing the severity of the problems where housemaids were forced into sex before they intend to do so. This finding is consistent with the finding of the study conducted in in three low income urban areas of Ethiopia where by 30.3 % of sexually active housemaids described their first sex as occurring under coercive conditions(9).

This study showed that, younger housemaids were more likely to be sexually violated than those housemaids who are in the older age groups (above 20 years). This finding was also consistent with the study from Rwanda (12). The possible explanation for this finding could be younger housemaids are physically as well as psychologically immature so that they are inherently manipulated easily and may not experience enough to be able to enforce clear boundaries for sexual activities and have lesser ability to object undesirable conditions. Moreover, the low maturity level may also hinder them from realizing warning signs early and the perpetrator who perceive that younger housemaids would show little or no resistance to their sexual advances may target these girls more readily.

Alcohol ever use was another factor associated with sexual violence among housemaids. From the result, housemaids who ever used alcohol were almost seven times more likely to be sexually violated than those who never tried alcohol drinking. This finding is also in line with the study

~~conducted among housemaids in Hawassa city(26) and Addis Ababa(21) .The possible explanation for this finding is that Consuming alcohol makes it more difficult for housemaids to protect themselves by interpreting and effectively acting on warning signs.~~

~~In this study involvement in a relationship was found to be significantly associated with life time sexual violence. House maids who were not in a union/married were found to be 72% less likely to face sexual violence as compared to those who are either married or are in union. The possible suggestion might be those housemaids who have boyfriend or husband might be less likely to say no for the fear of friendship discontinuation.~~

~~The housemaids original area of residence was another factor which showed significant association with life time sexual violence among the study participants. In this regard housemaids coming from rural areas were almost 2 times more likely to be sexually violated as compared to those from urban areas. The possible explanation for this finding could be housemaids from rural areas may be less exposed to sexual and reproductive health information because medias are not easily accessible in rural areas besides when they migrated to urban areas they will be culturally new for the locality and may not speak the local language so they may face cultural and communication barriers that makes them prone to sexual violence than other urban grown housemaids.~~

~~With respect to the year of experience as a housemaid, those housemaids who worked for less than five years were 3 times more likely to be sexually violated as compared to those housemaids who worked for more than five years. This result might be due to the fact that house maids who face any kind of sexual violence were less likely to remain in the sector and more likely to change their job to escape such abusive work environment. This idea is supported by the finding of the study conducted among out of school girls in six Ethiopian regions by population council(17). On the other hand housemaids who face sexual violence may also be fired by female or male employer to hide the situation and to keep their names so that the likelihood of remaining in the sector may be low. Also those housemaids who didn't face sexual violence are more likely to remain and continue working as a housemaid and may have a life experience of how to protect themselves from being sexually violated.~~ This study assessed the prevalence of sexual violence and associated phenomena among housemaids attending evening schools in urban settings in the Gedeo Zone. The results indicate that the lifetime prevalence of sexual violence among the housemaids is 60.2% (95% CI: 55.3%–64.5%). Notably, 52.8% of the incidents occurred within the past year. The level of sexual

violence among the subjects of this study represents a significant public health concern, and it reflects similar findings in a study conducted in Hosaena, in which 57.75% of the housemaids had faced sexual violence (24). An even greater incidence has been found in studies conducted in larger cities in Ethiopia, including Harar (72%) (7), Addis Ababa (76%) (25), and Mekele (84%) (13). This discrepancy mainly reflects the different contexts of the studies, in which there were larger numbers of the target population in various Ethiopian cities with different cultural and socioeconomic characteristics.

The most common type of sexual violence was sexual harassment, which was reported by 230 (58.4%) of the study's participants, supporting the findings of the study in Hosaena (24). This demonstrates that harassment can occur in numerous ways in diverse social settings. The lifetime prevalence of completed and attempted rape among the study participants was 11.2% and 19.0%, respectively, and the findings of studies from Hawassa (26) and Rwanda (12) reveal comparable levels of rape and attempted rape. Smaller percentages of such incidents were reported in studies from Harar (7), Addis Ababa (21, 25) and Mekelle (13). The extent of diverse types of sexual violence among the study participants in all these contexts and settings indicates a need for broad action to end violence against women, which remains a considerable obstacle to the achievement of women's and girls' human rights and the 2030 Agenda for Sustainable Development.

Another interesting finding regarding sexual violence among housemaids concerns the condition of their first sexual contact. In this study, 26.1% of the housemaid students had been sexually active, and 37.9% of them were coerced into their first sexual act, revealing the severity of a problem that results in a significant number of housemaids being forced into sex before they intend to do so. This finding is consistent with that of a study conducted in three low-income urban areas in Ethiopia, in which 30.3% of sexually active housemaids described their first sexual experience as occurring under coercive conditions (9).

This study reveals that younger housemaids were more likely to be sexually violated than older ones (above 20 years), a finding consistent with that of the study from Rwanda (12). Younger housemaids are physically as well as psychologically immature and thus more easily manipulated; they may have insufficient experience to enforce clear boundaries for sexual activities and may be less able to object to unwanted advances. Moreover, their lack of maturity may hinder their

recognizing of warning signs early, leading perpetrators to more readily target younger housemaids who offer little or no resistance to their sexual predation.

Alcohol use was another factor associated with sexual violence among housemaids. The results show that housemaids who had ever used alcohol were almost seven times more likely to be sexually violated than those who had never drunk alcohol. This finding is in line with the studies conducted among housemaids in Hawassa (26, 27) and Addis Ababa (21). Apparently, consuming alcohol makes it more difficult for housemaids to protect themselves by interpreting and effectively responding to warning signs.

In this study, involvement in a relationship was also significantly associated with lifetime sexual violence. Housemaids who were not in a union or married were 72% less likely to face sexual violence than those who were either married or in a union, suggesting that the main perpetrators of sexual violence were intimate partners. Housemaids with a boyfriend or husband may be less likely to resist attempts at sexual violence for fear that doing so would end the relationship.

The study participants' original area of residence was also significantly associated with lifetime sexual violence; housemaids from rural areas were almost twice as likely to be sexually violated as those from urban areas. Housemaids from rural areas are less exposed to sexual and reproductive health information because media are not easily accessible in those areas. Additionally, they experience cultural alienation when they migrate to urban areas and may not speak the local language, causing them to face cultural and communication barriers that make them more vulnerable to sexual violence than housemaids from urban areas.

Regarding years of experience as a housemaid, those who had worked for less than five years were three times more likely to be sexually violated than those who had worked for more than five years. This is explained by the fact that housemaids who face any kind of sexual violence are less likely to remain in the sector and more likely to seek different employment to escape an abusive work environment. This conclusion is supported by the finding of a study conducted among out-of-school girls in six Ethiopian regions by the population council (17). Additionally, housemaids who face sexual violence may be fired by female or male employers to conceal the matter and protect the employer's reputation, reducing the likelihood of a housemaid's remaining in the sector. Housemaids who do not face sexual violence are more likely to continue working as a housemaid and may have sufficient life experience to protect themselves from being sexually violated.

Formatted: Space After: 0 pt

Formatted: Font: (Default) +Body (Calibri), 11 pt, Not Italic

Father's level of educational attainment was AAanother factor that showed significantsignificantly associationed with life time sexual violence among housemaids. was level of fathers' education. In this regard, those housemaids whose fathers didn't have formal education were more than three times more likely to face sexual violence as compared to those whose fathers educational level was above secondary level. This may beis possibly due to the fact that those house-maids whose fathers were educated will get information about sexual matters, about their rights, how to protect themselves from being sexually violated and where to go in case of difficulties from their father before entering to domestic work service.

Strength and limitation of the study

Formatted: Font: 16 pt

This study was conducted to address the marginalized, vulnerable and the most neglected group of population with respect to sexual violence. However, important limitations are were admitted in this study. The main limitation of this study is was the fact that it onlyconfinement to involved school attending housemaids which might not reflect the reality among the general population of all housemaids found in the sector. There was also serious lack limitation of similar studies in our countryliteratures in the area to make more comparative conclusive discussion.

Conclusion and recommendation

Formatted: Font: 18 pt

This study showed thatIn the current study, sexual violence was found to be a major problem among going housemaids evening school female students who were working as a domestic worker. Nearly two-third of the students participants have were experiencing faced at least one form of sexual violence. The life--time completed rape and attempted rape level were also substantially considerable. Younger ages, housemaids who are in union, less experienced housemaids, those residing in rural area prior to the current present place of work, and those whose their parent, particularly father was less educated were more likely to experience sexual violence in this study. Thus, pragmatic intervention programs aiming to solve the problem of such vulnerable segment of populations needs to be in place. Government should focus this group particularly, with respect to labor laws and regulations to insure their basic human right. Further -evidence generating studies focusing on effective interventions modalities to address this population should be taken in to consideration by researchers.

Ethical Consideration

Formatted: Font: 18 pt

Formatted: Normal

Ethical clearance was obtained from Institutional Review Board of Dilla University, College of Health Science and Medicine. Written informed consent was obtained from each respondent after detailed explanation of the nature and procedure of the study. The information given by each respondent was kept with strict confidentiality and name was not recorded.

Formatted: Font: 18 pt, Bold

Funding

Formatted: Font: (Default) Times New Roman, 18 pt, Bold

Formatted: Font: (Default) Times New Roman, 14 pt

Funding for this study was covered by Dilla University NORHED/SENUPH project and the funding organization have no any role in methodology, analysis, interpretation, and write up of the manuscript.

Data Availability

Formatted: Font: 18 pt, Bold

Formatted: Font: 18 pt

Data underlying this study is readily available in Dryad public data repository with DOI https://doi.org/10.5061/dryad.hhmgqnkfv

Acknowledgement

Formatted: Font: 18 pt

We would like to acknowledge NORHED project for financial coverage Gedeo zone for their cooperation during conduction of the project. We are also very grateful to study participants and data collectors participated in the study.

REFERENCE

1.World health organization (WHO). Violence against women: intimate partner and sexual violence against women, 2014.

2.Human Rights Watch, Swept under the rug: abuses against domestic workers around the world, Human Rights Watch. 2006. Vol. 18, No. 7(C). 2006.

3.Internationa labour organization (ILO).Decent work for domestic workers. Report IV(1), fourth item on the agenda for (2)International Labour Conference, 99th session 2010.

4.Tadele, F., Pankhurst, A., Bevan, P., & Lavers, T. Migration and rural- urban linkages in Ethiopia and the implications for policy and development practice: Case studies of five rural and two urban sites in Addis Ababa, Amhara, Oromia and SNNP Regions. (Prepared for Irish Aid Ethiopia,2006.

5.Gebre KM. Vulnerability, Legal Protection and Work Conditions of Domestic Workers in Addis Ababa Human Right, Development and Social Justice 2012

6.Gebremedhin, M. M.. Procrastination in Recognizing the Rights of Domestic Workers in Ethiopia. Mizan Law Review, 2016 10(1), 38. https://doi.org/10.4314/mlr.v10i1.2.

7.Mustefa A. Sexual Violence against Women Domestic Worker Clients at Harar Higher Sexual and Reproductive Health Model Clinic of Family Guidance Association of Ethiopia, Eastern Ethiopia MSW Dissertation Research Project Report(MSWP - 001) , August 2014.

8.Biadegilegne, E.. Condition of work for adult female live-in paid domestic workers in Addis Ababa, Ethiopia,. 2011Kimmage DSC, Development Studies Centre.

9.Erulkar A and Ferede A. Social exclusion and early or unwanted sexual initiation among poor urban females in Ethiopia. International Perspec¬tives on Sexual and Reproductive Health,2009. 35(4): 186–193.).

10.Tobarak K and Rashid S.Violence against Women: Nature, Causes and Dimensions in Contemporary Bangladesh, Bangladesh e-Journal of Sociology.2013 10(1)79 ,,.

11.Aynalem s, Causes and Consequences of Sexual Abuse and Resilience Factors in Housemaids Working in Addis Ababa: A Qualitative Inquiry . The Ethiopian Journal of Social Sciences 2015 .Volume 1, Number 1,.

12.CLADHO and IDAY-International .Domestic workers' baseline survey focusing on child domestic workers and employers in Rwanda Kigali, July 2015

13. Gebremariam L, Tukue D, Desaleg S et al. Prevalence of Sexual Violence and its Associated Factors among Female Night Time Students of Junior and Secondary Schools in Mekelle City, Northern Ethiopia, Journal of Medical Science and Technology (RRJoMST). 2015; 4(2): 6–20p.

14.Asfaw M .Sexual Violence and its Consequence among Female Night School Students In Hawassa town, Southern Ethiopia: A cross-sectional study. 2010.

15.Belete Y M. challenges and opportunities of female domestic workers in accesing education a qualitative study from Bahir Dar city administration ,Amhara region ,Ethiopia April 2014.

16. Altinyelken .HK & Mat ,LM Sexual violence, schooling and silence: teacher narratives from a secondary school in Ethiopia, Compare: A Journal of Comparative and International Education .2018.48:4, 648-664, DOI: 10.1080/03057925.2017.1332517.

17.Erulkar, A., G. Medhin, and L. Negeri. .The Journey of Out-of-School Girls in Ethiopia: Examining Migration, Livelihoods, and HIV.2017 Addis Ababa: Population Council. .

18.Gedeo zone health office annual plan & achievement, 2011 E.C.

19.Statistical surveys Department of Economic and Social Affairs Statistics Division ,Guidelines for Producing Statistics on Violence against Women— United NationsNew York, 2014.

20.Garcia-Moreno C et al. WHO multi-country study on women's health and domestic violence against women: initial results on prevalence, health outcomes and women's responses. Geneva, World Health Organization, 2005.

21.Getachew M .Prevalence and determinants of sexual violence among female housemaids in selected junior secondary night school:cross sectional study Addis Ababa, Ethiopia ,2015.

22.DRUG and ALCHOL service south AustraliaASSIST for young people aged 15-17 draft clinical form ,2011.

23.Dilla town first stage court house rape victims of 2011 Ethiopia calander individual folders

24.Tagesse L ,Dubey R, Forms of Violence against Female Domestic Workers :The Case of Hossana Town, Hadiyya Zone SNNPR, Ethiopia Imperial Journal of Interdisciplinary Research (IJIR) .2017 Vol-3, Issue-8, ISSN: 2454-1362,.

25.Getachew Y. Addis Ababa University of Graduate studies Cross sectional assessment of violence against female domestic workers in gulele sub-city for local level intervention 2006.

26.Esatu F Sexual violence among house maids and its adverse reproductive health outcomes in Hawassa, Ethiopia an open access journal Volume 8, Issue 6 (Suppl).2017. ISSN: 2155-6113.

27. Yohannes K, Abebe L, Kisi T, Demeke W, Yimer S, Feyisso M, et al. The prevalence and predictors of domestic violence among pregnant women in Southeast Oromia, Ethiopia. Reprod Health 2019;16. https://doi.org/10.1186/s12978-019-0694-9.

Rebuttal Letter_Response to reviewers

PONE-D-20-12624
Prevalence of sexual violence and its associated factors among housemaids attending evening schools in urban setups of Gedeo zone, Southern Ethiopia: A school based cross sectional study
PLOS ONE
2. Please address the following:
- Please include additional information regarding the interview guide used in the study and ensure that you have provided sufficient details that others could replicate the analyses. For instance, if you developed a guide as part of this study and it is not under a copyright more restrictive than CC-BY, please include a copy, in both the original language and English, as Supporting Information. In addition, please include the details of the pre-testing of this tool, i.e. the number of participants and where they were recruited from.
- Please refrain from stating p values as 0.00, either report the exact value or employ the format p<0.001.

Response: The detail about pretest was explained in the manuscript page four paragraph one. Additional information about the tool was included and due to copyright issue we couldn't include as supporting information. P-value was corrected accordingly.

3. You indicated that you had ethical approval for your study. In your Methods section, please ensure you have also stated whether you obtained consent from parents or guardians of the minors included in the study or whether the research ethics committee or IRB specifically waived the need for their consent.

Response: It is explained in the manuscript under Ethical consideration section, page 6, Paragraph 1.

4. We note that you have indicated that data from this study are available upon request. PLOS only allows data to be available upon request if there are legal or ethical restrictions on sharing data publicly. For information on unacceptable data access restrictions, please see http://journals.plos.org/plosone/s/data-availability#loc-unacceptable-data-access-restrictions.

In your revised cover letter, please address the following prompts:

a) If there are ethical or legal restrictions on sharing a de-identified data set, please explain them in detail (e.g., data contain potentially identifying or sensitive patient information) and who has imposed them (e.g., an ethics committee). Please also provide contact information for a data access committee, ethics committee, or other institutional body to which data requests may be sent.

b) If there are no restrictions, please upload the minimal anonymized data set necessary to replicate your study findings as either Supporting Information files or to a stable, public repository and provide us with the relevant URLs, DOIs, or accession numbers. Please see http://www.bmj.com/content/340/bmj.c181.long for guidelines on how to de-identify and prepare

clinical data for publication. For a list of acceptable repositories, please see http://journals.plos.org/plosone/s/data-availability#loc-recommended-repositories.

We will update your Data Availability statement on your behalf to reflect the information you provide.

Response: We have uploaded the data on Dryad with DOI of https://doi.org/10.5061/dryad.hhmgqnkfv

5. Your ethics statement should only appear in the Methods section of your manuscript. If your ethics statement is written in any section besides the Methods, please delete it from any other section.

Response: Corrected accordingly. Page 6 paragraph 1

6. Please ensure that you refer to Figure 1 in your text as, if accepted, production will need this reference to link the reader to the figure.

Response: Corrected. Page 8

7. We note you have included a table to which you do not refer in the text of your manuscript. Please ensure that you refer to Table 1 and Table 2 in your text; if accepted, production will need this reference to link the reader to the Table.

Response: We have corrected the typing error regarding the table numbers

8. Please include a copy of Table 3 which you refer to in your text on page 6.

Reviewers' comments:

Reviewer's Responses to Questions

Comments to the Author

Reviewer #1: Many expressions of the author are not standard, including the display of the table. For example, when the author expresses the result, sometimes the numbers are expressed in Arabic numerals, sometimes in words, and some sentences are not clear. Please refer to the published article format and expression for revision.

Response: We have tried to make the presentation of the table to be to the standard. Unnecessary, numerical presentations in the table were modified

Reviewer #2: Does the manuscript adhere to the PLOS Data Policy? Additional details can be found at http://journals.plos.org/plosone/s/materials-and-software-sharing. (Answer options: Yes, No

"I don’t know"

1. It is obvious that the writing need to be improved. I suggest the authors ask for a help from someone whose native language is English.

Response: The manuscript English writing was proofread by Proffessional proofreaders to improve readability.

2. Section of “sample technique”:

1) The author described that a random cluster sampling was used to choose the evening school and then assigned the study participants according to the class grades. We didn’t find the class distribution of participants. Among the participants, “279 (70.8%) were adolescents (15-19 years old) whereas 115 (29.2%) were young adults (20- 24 years)”. Does the unbalanced sample exist

Response: Class distribution of the students was briefly explained in the methodology under sampling technique subsection page 4. Regarding issue about existence of unbalanced sampling, the proportion is not due to unbalanced sampling. It entirely due to the actual age distribution of the population.

2) “random sampling technique was used”, “data collectors were revisited the class room two times at different time intervals and for those who were absent after two revisits, the participant was replaced by another in the sampling frame”. How many women were replaced? Does the replace strategy have any effects on the study results?

Response: The number of women who replaced for their absence was not significant where only three women were missed and replaced. Replacement was also made by randomly selecting the participant from the previously unselected eligible population.

3. Section of “measurement”

1) The sexual harassment is the most common of sexual violence experience for housemaid have experienced in this study. The author didn’t explain sexual harassment in detail, has one appropriate joke related to sex been included? If this, the proportion of sexual violence might be overestimated.

Response: Explained under measurement subsection second paragraph page 5

2) The alcoholic use has not been evaluated semi-quantitatively or quantitatively. This is a common-sense question: What time and how much of the alcohols consumption is the most important for the victim suffer from sexual violence.

Response: The ASSIST tool is standard tool for assessment of the common substance uses based on the frequency and duration of use. We used that tool. With that tool we couldn’t find any women fulfilling criteria for substance use. So we measure the episodes of occasional use and classified alcohol use as ever use or not at all

4. Section of “results”:

1) In the descriptive analysis section, the author only needs to reveal the proportion of some important variables, for example those covariates lead to significant changes in the logistic model or other characteristic variables related to sexual violent founded from previous study.

Response: We have tried to remove some variables thought to be less important to present in this study. However, as one of the objective of this study is to describe the violence and the characteristics of the study participants, we retained most of the variables thought to have relevant descriptive information.

2) Are the forms of the tables' correct? Please refer to the author guidance of PLOS ONE.

5. Section of "Discussion"

1) The author discussed the results in comparability instead of analysis.

Response: We have tried to present with the analysis of the findings

2) "This might be due to…", "The possible explanation for this finding…", "The possible suggestion might be…", "This result might be due to …" were used by authors to explain the association between the related factors and sexual violence. It is suspicious that the author's knowledge for the study field or the amounts of reference the author has read.

Response: We have modified our way of explanation accordingly

3) It is interesting for the findings that the educational attainments of fathers were associated with the sexual violence for the housemaids suffered. A health education system should be considered to help establishing an intimate father-daughter relationship in these districts.

Response: The finding of the study was disseminated to the local health planners and this will be an input to address the issue.

Thank you for your interesting and valuable comments that made us to improve our work substantially.
