# Supplementary material for: Prevalence of sexual violence and its associated factors among housemaids attending evening schools in urban settings of Gedeo zone, Southern Ethiopia: A school based cross sectional study
Source: PLoS One. 2021 Oct 29;16(10):e0258953. doi: 10.1371/journal.pone.0258953 (PMC8555818; doi:10.1371/journal.pone.0258953)
Supplement: Attachment — Submitted filename: Response to reviewers.docx [file pone.0258953.s001.docx]

**Rebuttal Letter_Response to reviewers**

PONE-D-20-12624
Prevalence of sexual violence and its associated factors among housemaids attending evening schools in urban setups of Gedeo zone, Southern Ethiopia: A school based cross sectional study
PLOS ONE
2. Please address the following:
- Please include additional information regarding the interview guide used in the study and ensure that you have provided sufficient details that others could replicate the analyses. For instance, if you developed a guide as part of this study and it is not under a copyright more restrictive than CC-BY, please include a copy, in both the original language and English, as Supporting Information. In addition, please include the details of the pre-testing of this tool, i.e. the number of participants and where they were recruited from.
- Please refrain from stating p values as 0.00, either report the exact value or employ the format p<0.001.

 Response: The detail about pretest was explained in the manuscript page four paragraph one. Additional information about the tool was included and due to copyright issue we couldn’t include as supporting information. P-value was corrected accordingly.

3. You indicated that you had ethical approval for your study. In your Methods section, please ensure you have also stated whether you obtained consent from parents or guardians of the minors included in the study or whether the research ethics committee or IRB specifically waived the need for their consent.

 Response: It is explained in the manuscript under Ethical consideration section, page 6, Paragraph 1.

4. We note that you have indicated that data from this study are available upon request. PLOS only allows data to be available upon request if there are legal or ethical restrictions on sharing data publicly. For information on unacceptable data access restrictions, please see http://journals.plos.org/plosone/s/data-availability#loc-unacceptable-data-access-restrictions.

 In your revised cover letter, please address the following prompts:

a) If there are ethical or legal restrictions on sharing a de-identified data set, please explain them in detail (e.g., data contain potentially identifying or sensitive patient information) and who has imposed them (e.g., an ethics committee). Please also provide contact information for a data access committee, ethics committee, or other institutional body to which data requests may be sent.

b) If there are no restrictions, please upload the minimal anonymized data set necessary to replicate your study findings as either Supporting Information files or to a stable, public repository and provide us with the relevant URLs, DOIs, or accession numbers. Please see http://www.bmj.com/content/340/bmj.c181.long for guidelines on how to de-identify and prepare clinical data for publication. For a list of acceptable repositories, please see http://journals.plos.org/plosone/s/data-availability#loc-recommended-repositories.

We will update your Data Availability statement on your behalf to reflect the information you provide.

 Response: We have uploaded the data on Dryad with DOI of <https://doi.org/10.5061/dryad.hhmgqnkfv>

5. Your ethics statement should only appear in the Methods section of your manuscript. If your ethics statement is written in any section besides the Methods, please delete it from any other section.

 Response: Corrected accordingly. Page 6 paragraph 1

6. Please ensure that you refer to Figure 1 in your text as, if accepted, production will need this reference to link the reader to the figure.

 Response: Corrected. Page 8

7. We note you have included a table to which you do not refer in the text of your manuscript. Please ensure that you refer to Table 1 and Table 2 in your text; if accepted, production will need this reference to link the reader to the Table.

 Response: We have corrected the typing error regarding the table numbers

8. Please include a copy of Table 3 which you refer to in your text on page 6.


Reviewers' comments:

Reviewer's Responses to Questions

**Comments to the Author**

Reviewer #1: Many expressions of the author are not standard, including the display of the table. For example, when the author expresses the result, sometimes the numbers are expressed in Arabic numerals, sometimes in words, and some sentences are not clear. Please refer to the published article format and expression for revision.

Response: We have tried to make the presentation of the table to be to the standard. Unnecessary, numerical presentations in the table were modified

Reviewer #2: Does the manuscript adhere to the PLOS Data Policy? Additional details can be found at http://journals.plos.org/plosone/s/materials-and-software-sharing. (Answer options: Yes, No

"I don’t know"

1. It is obvious that the writing need to be improved. I suggest the authors ask for a help from someone whose native language is English.

Response: The manuscript English writing was proofread by Proffessional proofreaders to improve readability.
2. Section of “sample technique”:
1) The author described that a random cluster sampling was used to choose the evening school and then assigned the study participants according to the class grades. We didn’t find the class distribution of participants. Among the participants, “279 (70.8%) were adolescents (15-19 years old) whereas 115 (29.2%) were young adults (20- 24 years)”. Does the unbalanced sample exist

Response: Class distribution of the students was briefly explained in the methodology under sampling technique subsection page 4. Regarding issue about existence of unbalanced sampling, the proportion is not due to unbalanced sampling. It entirely due to the actual age distribution of the population.

2) “random sampling technique was used”, “data collectors were revisited the class room two times at different time intervals and for those who were absent after two revisits, the participant was replaced by another in the sampling frame”. How many women were replaced? Does the replace strategy have any effects on the study results?

Response: The number of women who replaced for their absence was not significant where only three women were missed and replaced. Replacement was also made by randomly selecting the participant from the previously unselected eligible population.

3. Section of “measurement”
1) The sexual harassment is the most common of sexual violence experience for housemaid have experienced in this study. The author didn’t explain sexual harassment in detail, has one appropriate joke related to sex been included? If this, the proportion of sexual violence might be overestimated.

Response: Explained under measurement subsection second paragraph page 5

2) The alcoholic use has not been evaluated semi-quantitatively or quantitatively. This is a common-sense question: What time and how much of the alcohols consumption is the most important for the victim suffer from sexual violence.

Response: The ASSIST tool is standard tool for assessment of the common substance uses based on the frequency and duration of use. We used that tool. With that tool we couldn’t find any women fulfilling criteria for substance use. So we measure the episodes of occasional use and classified alcohol use as ever use or not at all

4. Section of “results”:
1) In the descriptive analysis section, the author only needs to reveal the proportion of some important variables, for example those covariates lead to significant changes in the logistic model or other characteristic variables related to sexual violent founded from previous study.

Response: We have tried to remove some variables thought to be less important to present in this study. However, as one of the objective of this study is to describe the violence and the characteristics of the study participants, we retained most of the variables thought to have relevant descriptive information.
2) Are the forms of the tables’ correct? Please refer to the author guidance of PLOS ONE.

5. Section of “Discussion”
1) The author discussed the results in comparability instead of analysis.

Response: We have tried to present with the analysis of the findings
2) “This might be due to…”, “The possible explanation for this finding…”, “The possible suggestion might be…”, “This result might be due to …” were used by authors to explain the association between the related factors and sexual violence. It is suspicious that the author’s knowledge for the study field or the amounts of reference the author has read.

Response: We have modified our way of explanation accordingly
3) It is interesting for the findings that the educational attainments of fathers were associated with the sexual violence for the housemaids suffered. A health education system should be considered to help establishing an intimate father-daughter relationship in these districts.

Response: The finding of the study was disseminated to the local health planners and this will be an input to address the issue.

**Thank you for your interesting and valuable comments that made us to improve our work substantially.**
