# Supplementary material for: Prevalence of sexual violence and its associated factors among housemaids attending evening schools in urban settings of Gedeo zone, Southern Ethiopia: A school based cross sectional study
Source: PLoS One. 2021 Oct 29;16(10):e0258953. doi: 10.1371/journal.pone.0258953 (PMC8555818; doi:10.1371/journal.pone.0258953)
Supplement: Attachment — Submitted filename: Response to reviewers.docx [file pone.0258953.s003.docx]

**Response to reviewers**

- The authors used the face to face interview how did the participants feel comfortable such a sensitive issue? How did they prevent the bias and protect the correct of the answer?
  - *From the report of the data collectors during the time of pre-test, the feeling of the respondents for the questions was not embarrassing and the respondents were very comfortable with the approach. The same was reported form the daily routine follow up of actual data collection*
  - *The data collectors were well trained to handle such issue*
  - *The participants were adequately reassured for the confidentiality of the information they provide*
  - *The interview was conducted at isolated place with adequate privacy*
- Why did the author create the survey English language? Are these instruments available in their language, if not, did you make content and language validity of them?

*We have used the survey tool that was used previously in comparable settings. We couldn’t find the tool in local language. We have tried to familiarize it with the context through pre-test and training of the surveyors.*

- Ethical Consideration
  The main concern has protected minors, how did you protect them, when they disclosure sexual violence. What is your legal responsibility for the researcher? Is it any mandatory report about disclosure about SV. Please give your legal policy about this concern.

*Confidentiality of the response was highly secured. As a researcher, we have provided contact information for any possible risk associated with the disclosure information during data collection. The respondents were advised for possible legal body and regulation regarding sexual violence according to guiding principle of acting in the best interests of the child. Otherwise, the right to lodge a complaint shall be exercised only by legal representative according to regulation in the locality and most of the participants of our study were older children (>15 years) and there were no issues regarding capacity to consent or a relationship of power as of our data collectors report from day to day follow up.*

Discussion

- We have revised the discussion according to your suggestion including discussion of findings that were missed in the previous revision

Thank you for your constructive comments. We are really appreciate the contribution you made to the improvement of our work.

Mohammed Feyisso Shaka

Kalkidan Gezahagn

Selamawith Semagn
